# Analysis of the Yeast Peptidome and Comparison with the Human Peptidome

**DOI:** 10.1371/journal.pone.0163312

**Published:** 2016-09-29

**Authors:** Sayani Dasgupta, Ciyu Yang, Leandro M. Castro, Alexandre K. Tashima, Emer S. Ferro, Robyn D. Moir, Ian M. Willis, Lloyd D. Fricker

**Affiliations:** 1 Department of Molecular Pharmacology, Albert Einstein College of Medicine, Bronx, New York, 10461, United States of America; 2 Department of Pathology, Memorial Sloan Kettering Cancer Center, New York, New York, 10065, United States of America; 3 Biomedical Science Institute, Campus on the São Paulo Coast, São Paulo State University, São Vicente, 11330–900, SP, Brazil; 4 Department of Biochemistry, Escola Paulista de Medicina, Federal University of Sao Paulo, Sao Paulo, SP, 04023–901, SP, Brazil; 5 Department of Pharmacology, Biomedical Science Institute, University of São Paulo, São Paulo, 05508–000, SP, Brazil; 6 Department of Biochemistry, Albert Einstein College of Medicine, Bronx, New York, 10461, United States of America; 7 Department of Systems & Computational Biology, Albert Einstein College of Medicine, Bronx, New York, 10461, United States of America; 8 Department of Neuroscience, Albert Einstein College of Medicine, Bronx, New York, 10461, United States of America; University of Florida, UNITED STATES

## Abstract

Peptides function as signaling molecules in species as diverse as humans and yeast. Mass spectrometry-based peptidomics techniques provide a relatively unbiased method to assess the peptidome of biological samples. In the present study, we used a quantitative peptidomic technique to characterize the peptidome of the yeast *Saccharomyces cerevisiae* and compare it to the peptidomes of mammalian cell lines and tissues. Altogether, 297 yeast peptides derived from 75 proteins were identified. The yeast peptides are similar to those of the human peptidome in average size and amino acid composition. Inhibition of proteasome activity with either bortezomib or epoxomicin led to decreased levels of some yeast peptides, suggesting that these peptides are generated by the proteasome. Approximately 30% of the yeast peptides correspond to the N- or C-terminus of the protein; the human peptidome is also highly represented in N- or C-terminal protein fragments. Most yeast and humans peptides are derived from a subset of abundant proteins, many with functions involving cellular metabolism or protein synthesis and folding. Of the 75 yeast proteins that give rise to peptides, 24 have orthologs that give rise to human and/or mouse peptides and for some, the same region of the proteins are found in the human, mouse, and yeast peptidomes. Taken together, these results support the hypothesis that intracellular peptides may have specific and conserved biological functions.

## Introduction

Peptides perform a number of diverse functions in organisms from yeast to humans. Many chemical intercellular messengers are peptides, such as mammalian peptide hormones and neuropeptides [1,2]. Some species of yeast also use peptides to signal as pheromones or mating signals, such as alpha-mating factor and a-mating factor of *Saccharomyces cerevisiae* [3,4]. In higher eukaryotes with a functional immune system (i.e. jawed vertebrates), peptides produced within the cell by proteasome-mediated cleavages are displayed on the cell surface attached to Major Histocompatibility Complex (MHC) proteins and function in immune system recognition [5–7]. Only peptides that are a specific length (typically 9–10 amino acids), contain certain sequence motifs, and which are transported into the lumen of the endoplasmic reticulum can potentially bind to MHC proteins; these represent an extremely small fraction of all proteasome-mediated cleavages, and the vast majority of proteasome products are thought to be rapidly degraded within seconds by cellular peptidases [8–10]. One of the arguments made to support the rapid degradation of intracellular peptides was that if these peptides were not removed, they would wreak havoc on cellular activity [8]. For example, synthetic peptides are widely used to disrupt protein function within cells [11,12], and endogenous peptides could potentially cause numerous effects if they were allowed to accumulate within cells. However, if the production and degradation of intracellular peptides was tightly controlled, these peptides could provide a mechanism for regulation of protein-protein interactions or other biochemical functions.

Peptides derived from intracellular proteins have been identified in a number of studies using mass spectrometry-based techniques [13–23]. Peptidomics studies on mouse brain identified approximately one thousand distinct peptides, with the majority derived from proteins normally present within the cytosol, mitochondria, or nucleus [16]. A recent study on rat hypothalamus detected over 16,000 unique peptides, and a large fraction were derived from intracellular proteins [23]. Peptidomics studies on human cell lines identified hundreds of peptides, nearly all of which were derived from intracellular proteins [17]. Some of the peptides detected in the human cell lines were derived from abundant proteins, but only a small subset of the most abundant proteins was reflected in the pool of detected peptides [17]. Levels of nearly all of the intracellular peptides in several different human and mouse cell lines were altered by treatment of cells with proteasome inhibitors, suggesting that these peptides are proteasome products [19–22]. The proteasome cleaves a typical protein into dozens of distinct peptides, but for most proteins only 1–2 peptide fragments were detected for each protein [17,19–22]. This is consistent with the idea that most, but not all, proteasome products are rapidly degraded by intracellular enzymes. Those peptides that are not rapidly degraded may be protected by interactions with cellular proteins, and these interactions may alter the function of the cellular proteins.

The catalytically active subunits of the proteasome, as well as most other components of the proteasome complex, are highly conserved between human and *Saccharomyces cerevisiae* [24,25]. This species of yeast has been very well studied in terms of mRNA levels, protein levels, protein synthesis rates, and protein degradation rates [26–33]. However, no previous study has examined the yeast peptidome. In the present study, we analyzed the peptidome of wild-type yeast. Because peptides in mammalian cell lines are affected by treatment with proteasome inhibitors, we tested whether proteasome inhibitors altered the levels of peptides in wild-type yeast as well as strains deleted for plasma membrane-bound multi-drug transporters (*PDR5* and *SNQ2*). To further explore the role of specific proteasome forms, we examined the levels of peptides in a yeast strain lacking the proteasome activator *BLM10*. The results of these analyses reveal striking similarities between the yeast and human peptidome, indicating that some properties of the peptidome have been highly conserved through evolution.

## Materials and Methods

### Materials

All reagents were purchased from Sigma unless otherwise indicated. Yeast nitrogen base with ammonium sulfate was obtained from MP Biomedicals. Dulbecco’s Phosphate Buffered Saline (DPBS) was obtained from Invitrogen. Bortezomib and epoxomicin were purchased from LC Laboratories and Millipore, respectively. Acetonitrile was obtained from Fisher. Hydrochloric acid, trifluoroacetic acid (TFA) mass spectroscopy grade and C-18 spin columns were purchased from Pierce Thermo Scientific. Succinyl-Leu-Leu-Val-Tyr-7-amino-4-methylcoumarin (Suc-Leu-Leu-Val-Tyr-AMC) was obtained from Bachem. The isotopic labeling reagents 4-trimethylammoniumbutyryl-N-hydroxysuccinimide (TMAB-NHS) containing either 0, 3, 6, or 9 atoms of deuterium (D0-, D3-, D6-, and D9-TMAB-NHS, respectively) were synthesized as described [34]. The *blm10*Δ::NatMX3 strain yMS63 [35] and its isogenic wild-type BY4741 parent MATa *his3*Δ1 *leu2*Δ0 *met15*Δ0 *ura3*Δ0 [36,37] were obtained from Marion Schmidt (Department of Biochemistry, Albert Einstein College of Medicine). The deletion strains *pdr5*Δ::KanMX4 and *snq2*Δ::KanMX4 in BY4741 were generated in the yeast deletion project [38].

### Yeast culture for peptidomic analyses

Yeast cells were grown at 30°C to early log phase (less than 1.0 OD600) in synthetic complete medium (0.67% yeast nitrogen base with ammonium sulfate and 2% glucose, supplemented with histidine, leucine, uracil and methionine). Cells were collected by centrifugation at 3,000 × g for 5 min, washed with DPBS, and recentrifuged. For some experiments, the pellet was resuspended in 80°C lysis buffer (50 mM NaH_2_PO_4_, 50 mM NaCl, and 50 mM MgCl_2_) at a volume of 0.4 × mass of the pellet and immediately incubated in an 80°C water bath for 20 min to inactivate proteases. Cell lysates were prepared by freezing in liquid nitrogen prior to breakage in a Retsch MM301 grinding mill according to the manufacturer’s instructions. In other experiments, the cell pellet was resuspended in 80°C water and incubated at this temperature for 20 min to inactivate proteases, cooled to room temperature, combined with glass beads and ground using a Mini-Beadbeater (BioSpec Products) for 8 cycles of 30 s each, with a 2 min incubation on ice between each cycle. There was no major difference in the peptide composition between these two cell breakage methods. The cell lysate was dissolved in water, cooled on ice for 10 min, and acidified with HCl to a final concentration of 10 mM. After 15 min incubation on ice, the suspension was centrifuged at 13,000 × g for 30 min at 4°C. The supernatant was removed, combined with 250 μL of dibasic sodium phosphate (0.4 M, pH 9.5) and stored at -80°C until labeling.

For proteasome inhibitor studies, yeast cells were grown to early log phase (less than 1.0 OD600), split into two equal volumes and treated with 1 μM or 10 μM bortezomib, 4 μM epoxomicin, or a comparable amount of DMSO (maximum 0.1%) for 1 hour. Independent duplicate cultures were grown to generate control and inhibitor-treated biological replicates, as shown in S1 Fig. The cells were collected by centrifugation at 3,000 × g for 5 min, washed with DPBS supplemented with the proteasome inhibitor at the same concentration as used for the treatment, and centrifuged again. Peptides were extracted from cell pellets as described above.

For the studies comparing the effect of the Blm10 proteasome cap on the cellular peptidome, the *blm10*Δ strain yMS63 and the isogenic wild-type strain BY4741 were used [36,37]. The growth conditions, harvesting, and peptide extraction were similar to that described above.

### Proteasome activity assay

Wildtype, *PDR5* and *SNQ2* gene deletion strains were grown to early log phase and treated with proteasome inhibitors as described above. The cells were collected by centrifugation at 3,000 × g for 5 min, followed by two washes with water. The pellet was resuspended in 1 ml water, ground with glass beads using a Mini-Beadbeater (BioSpec Products), and centrifuged at 13,000 × g for 5 min at 4°C. A fraction of the supernatant was incubated in 50 mM Tris HCl buffer, pH 7.5, containing 40 mM KCl, 5 mM MgCl_2_, 0.5 mM ATP, 1 mM DTT with 100 μM of the proteasome substrate Suc-Leu-Leu-Val-Tyr-AMC at 37°C for 1 hour. After incubation, proteasomal activity was quantified by fluorescence measurement of the substrate (380 nm excitation, 460 nm emission).

### Isotopic labeling

Quantitative peptidomics was performed using isotopic forms of trimethylammonium butyrate (TMAB) activated with N-hydroxysuccinimide (NHS), synthesized and used as described [34]. Each group within an experiment (control/treated) was labeled with a different isotopic tag, as shown in S1 Fig. The TMAB-NHS labels were dissolved in DMSO to a concentration of 400 μg/μL and 7.5 mg of label was used per sample. The pH of the peptide extract was adjusted to 9.5 with 1 M NaOH at the start of the experiment. Labeling was performed over 8 rounds; 2.3 μL of the label was added to the extract every 20 min. The pH was measured between each round and if necessary, brought back to 9.5 for the first five rounds. For rounds 6–8, the pH was not adjusted after the addition of the TMAB-NHS reagent. After the final round of labeling, the pH was adjusted to 9.5 again, extracts were incubated at room temperature for 90 min, following which 30 μL of 2.5 M glycine was added to quench unreacted label. After 40 min of incubation at room temperature, the labeled extracts for a single experiment were pooled and filtered through Amicon Ultracel-10 K units. 30 μL of a 2 M solution of hydroxylamine hydrochloride was added over three rounds to the pooled and filtered sample to hydrolyze any labeled tyrosines. The pH was measured after the addition of hydroxylamine and adjusted to 9.0 with 1 M NaOH. Samples were desalted through C-18 spin columns and peptides were eluted using 160 μL of 0.5% TFA and 70% acetonitrile. Samples were freeze-dried in a vacuum centrifuge and stored at −80°C until analysis by mass spectrometry.

### Mass spectrometry

The LC-MS/MS experiments were performed on a Synapt G2 mass spectrometer coupled to a nanoAcquity capillary liquid chromatography (LC) system (Waters, Milford, MA, USA). The peptide mixture was desalted online for 3 min at a flow rate of 5 μL/min of phase A (0.1% formic acid) using a Symmetry C18 trapping column (5-μm particles, 180-μm inner diameters, 20-mm length; Waters). The mixture of trapped peptides was subsequently separated by elution with a gradient of 7–65% of phase B (0.1% formic acid in acetonitrile) through a BEH 130 C18 column (1.7-μm particles, 75-μm inner diameters, 100-mm length; Waters) in 42 min. The data were acquired in the data-dependent mode and the MS spectra of multiple-charged protonated peptides generated by electrospray ionization were acquired for 0.2 s from m/z 300–1600. The three most intense ions exceeding base peak intensity threshold of 2500 counts were automatically mass selected and dissociated in MS/MS by 15- to 60-eV collisions with argon for 0.2 s. The typical LC and electrospray ionization conditions consisted of a flow rate of 250 nL/min, a capillary voltage of 3.0 kV, a block temperature of 70°C, and a cone voltage of 50 V. The dynamic peak exclusion window was set to 90 s.

### Quantification of relative peptide levels

The MS spectra were manually evaluated. The intensity of the monoisotopic peaks of peptide containing each of the isotopic labels was logged into a spreadsheet. If a peptide was found with multiple charge states, the peak intensity of each state was averaged to arrive at a single number for the relative level of each isotopically-tagged form. See S1 Fig for details on the labeling strategy and the number of replicates. Every experiment included two biological replicates of each treated and control group, as shown in S1 Fig.

### MS/MS identification of peptides

Peptides were identified by MS/MS sequencing using the Mascot program (Matrix Science) followed by manual verification of the spectra using MassLynx, version 4.0 (Waters). The database searched was SwissProt_AC_20150324, limited to yeast *Saccharomyces cerevisiae* (7,904 sequences). No cleavage site was specified, and variable post-translational modifications selected for the searches included the TMAB labels (termed ‘GIST’ in Mascot), N-terminal protein acetylation, methionine oxidation, and cyanylation of Cys. The Mascot results were manually evaluated to exclude false positives, based on previously established criteria [34,39,40]. These criteria are listed in S1 Appendix along with representative data showing the evaluation process for a peptide with a low Mascot score. S2 Appendix shows the evaluation of another peptide which failed to meet the criteria. Supplemental files show the sequences (including cleavage sites) for the yeast peptides (S1 Table) and their precursor proteins/genes, including Mascot scores (S2 Table). The mass spectrometry data for the yeast peptidomics analyses have been deposited to the ProteomeXchange Consortium [41] via the MassIVE partner repository (MassIVE ID: MSV000080119).

The human peptidome was compiled from previous studies on human cell lines HEK293T, SH-SY5Y, and MCF-7 cell lines [17,19–21,42]. In addition, recent peptidomic data using the RPMI-8226 cell line were included; these analyses were performed using identical procedures as those used for the other cell lines [17]. Peptides detected in untreated cells were included in the compiled human peptidome database; those peptides only detected in cells treated with proteasome inhibitors or other reagents were excluded from the database. Supplemental files show the sequences (including cleavage sites) for the human peptides (S3 Table) and their precursor proteins/genes (S4 Table). The mouse peptidome was previously reported [16].

## Results

**Fig 1:**
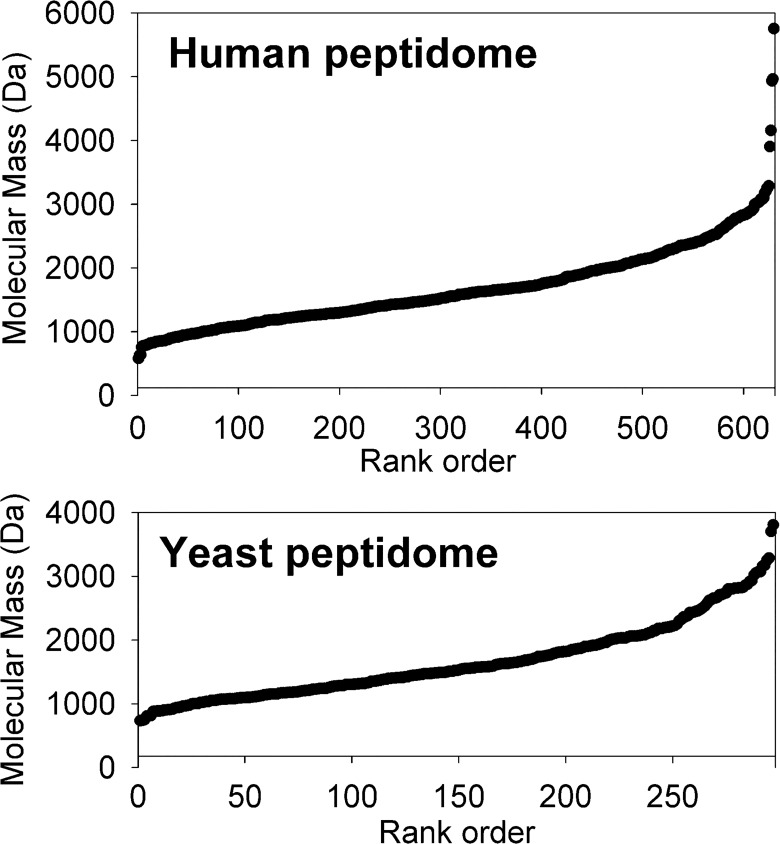
Comparison of the size distribution of peptides in human cell lines and yeast. The 627 peptides identified in human cell lines and the 297 peptides identified in yeast were each sorted by rank of the peptide’s molecular mass and plotted. For the human peptidome, the average mass was 1675 Da and the median was 1559 Da. For the yeast peptidome, the average mass was 1640 Da and the median was 1523 Da.

Altogether, 297 unique peptides were identified from MS/MS analysis of the 5 LC/MS runs performed in this study (S1 Table). Additional peptides were detected in the MS spectra but could not be identified, either because no MS/MS data were obtained or the quality of the MS/MS spectra were not sufficient to determine the peptide sequence. The 297 identified peptides were derived from 75 distinct proteins/genes (S2 Table). Approximately one half of the identified peptides arose from 8 proteins. The average mass of the yeast peptides is 1640 Da, the median is 1523 Da, and 90% of the peptides are between 900 and 3000 Da (Fig 1). The amino acid composition of the peptides shows the most abundant amino acid is Ala (10.6%), with Lys, Val, Leu, Gly, Asp, and Glu also abundant (Table 1). Cys is not detected in the peptidome, but this omission is likely to be an artifact of the extraction and/or labeling procedure [43]. Otherwise, the amino acid composition of the peptidome is generally similar to the amino acid composition of the intracellular and nuclear yeast proteome [44].

**Table 1:** Amino acid composition of peptides found in human cell lines and yeast cells.

	Human Peptides		Yeast Peptides		Yeast Proteins—Intracellular	Yeast Proteins—Nuclear
	#	%	#	%	% ± SD	% ± SD
A	866	9.1	471	10.6	7.9 ± 2.9	8.3 ± 5.0
C	3	0.0	0	0.0	1.9 ± 2.4	1.6 ± 1.7
D	645	6.8	335	7.5	5.5 ± 2.0	4.7 ± 2.6
E	690	7.2	322	7.2	7.1 ± 3.2	6.5 ± 3.6
F	376	3.9	135	3.0	3.9 ± 1.8	2.7 ± 1.7
G	787	8.3	342	7.7	7.1 ± 2.6	6.3 ± 2.8
H	137	1.4	73	1.6	2.1 ± 1.4	2.1 ± 1.4
I	502	5.3	235	5.3	5.2 ± 2.0	3.7 ± 2.0
K	696	7.3	392	8.8	6.7 ± 2.8	7.9 ± 6.5
L	900	9.4	359	8.1	8.6 ± 2.4	7.4 ± 3.2
M	164	1.7	40	0.9	2.4 ± 1.3	2.3 ± 1.2
N	309	3.2	202	4.5	4.0 ± 1.8	3.7 ± 2.2
P	560	5.9	225	5.1	5.3 ± 4.0	6.9 ± 3.4
Q	395	4.1	172	3.9	4.4 ± 3.0	4.7 ± 2.8
R	392	4.1	131	2.9	4.9 ± 2.2	8.7 ± 13.5
S	634	6.7	304	6.8	6.6 ± 2.0	8.8 ± 3.2
T	438	4.6	241	5.4	5.3 ± 1.7	5.1 ± 2.1
V	813	8.5	377	8.5	6.8 ± 1.9	5.3 ± 1.8
W	70	0.7	9	0.2	1.2 ± 0.8	0.7 ± 0.7
Y	156	1.6	87	2.0	3.1 ± 1.5	2.4 ± 1.3

SD, standard deviation. The amino acid composition of yeast proteins is from Cedano et al [44].

To compare the yeast peptidome with the human cell line peptidome, we compiled a master list of all peptides that were identified in peptidomics studies on HEK293T, SH-SY5Y, MCF7, and RPMI-8226 cells [17,19–21,42]. These previous studies included 83 LC/MS runs. Altogether, this combined dataset contains 627 distinct peptides (S3 Table) that are derived from 153 distinct proteins/genes (S4 Table). Additional human peptides were detected by MS analysis but their identities could not be determined from the MS/MS analysis. Thus, both yeast and human datasets reflect a subset of the total number of peptides detected by MS. Although a greater number of human peptides were identified than yeast peptides, this does not necessarily mean that human cells have more peptides than yeast; many more LC/MS runs of human peptides were analyzed and the total numbers of identified peptides cannot be compared between human and yeast.

The size distribution of the identified human peptides is very similar to that of yeast peptides, with an average mass of 1675 Da and median mass of 1559 Da for the human peptides (Fig 1). As with the yeast peptides, 90% of the identified human peptides were between 900 and 3000 Da (Fig 1). The amino acid composition of the human peptides is very similar to that of the yeast peptides (Table 1). Neither the yeast nor human peptides are enriched in residues such as Pro that are resistant to the common cellular aminopeptidases (Table 1). The most common N-terminal amino acid is Ala in both the yeast (14%) and human peptidome (16%). Other common N-terminal amino acids are Leu, Ile, Ser, Val, and Gly; these residues are present on the N-terminus of approximately half of the yeast and human peptides (S1 and S3 Tables). Only 9% of the yeast peptides and 21% of the human peptides have N-terminal acetyl groups. These numbers are consistent with the finding that 15% of the yeast peptides and 26% of the human peptides represent the N-terminal fragment of the protein (discussed below) and 57% of yeast proteins and 84% of human proteins are N-terminally acetylated [45]. Thus, the presence of N-terminal acetyl groups on the yeast and human peptides is a reflection of the proteins from which the peptides are derived, and does not deviate from the expected frequency.

**Fig 2:**
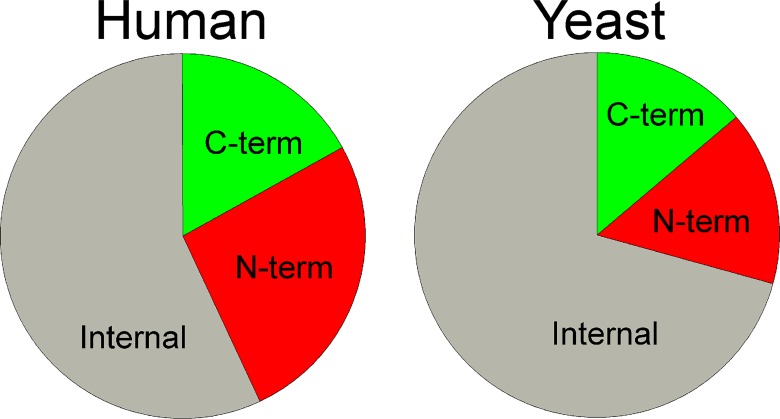
Location of peptides within proteins. C-term refers to peptides that contain the C-terminal residue of the protein. N-term includes peptides with the N-terminal initiation Met as well as peptides with this Met removed (including acetylated peptides). A small number of proteins are known to contain precursor sequences that are cleaved to produce the mature form of the protein, and several of the human N-terminal peptides correspond to the N-terminus of the mature form. See S1 and S3 Tables for peptide sequences.

A previous analysis of intracellular mouse brain peptides found that close to one half of the peptides represented the N- or C-terminus of the protein [16]. In contrast, proteomic studies that digest proteins with trypsin prior to MS analysis detect mainly peptides that are internal fragments of the protein [43]. Similar to the previous report on intracellular mouse peptides, 43% of the human cell line peptides represent either the N-terminus (26%) or C-terminus (17%) of the proteins from which the peptides are derived (Fig 2). The yeast peptidome also shows an abundance of peptides derived from the N-terminus (15%) and C-terminus (14%) of the proteins (Fig 2), relative to the predicted abundance of <1% based on consideration of the average size of the peptides and proteins from which they are produced.

The method used to extract peptides from yeast avoided hot acid which causes hydrolysis of Asp-Pro bonds [46]. None of the yeast peptides detected in our analysis resulted from cleavage at Asp-Pro sites. Pro was not very common in the P1 position of the cleavage site; only one yeast peptide and two human peptides resulted from cleavage of a Pro-Xaa bond. Acidic residues (Asp, Glu) were found in the P1 position of the cleavage site of 11% of the yeast peptides and 4% of the human peptides. Basic residues (Lys, Arg) were found in the P1 position of the cleavage site of 22% of the yeast peptides and 13% of the human peptides. Hydrophobic residues (Leu, Met, Val, Ala, Phe, Tyr, and Trp) were found in the P1 position of approximately one half of the cleavage sites for both yeast and human peptides (S1 and S3 Tables).

**Fig 3:**
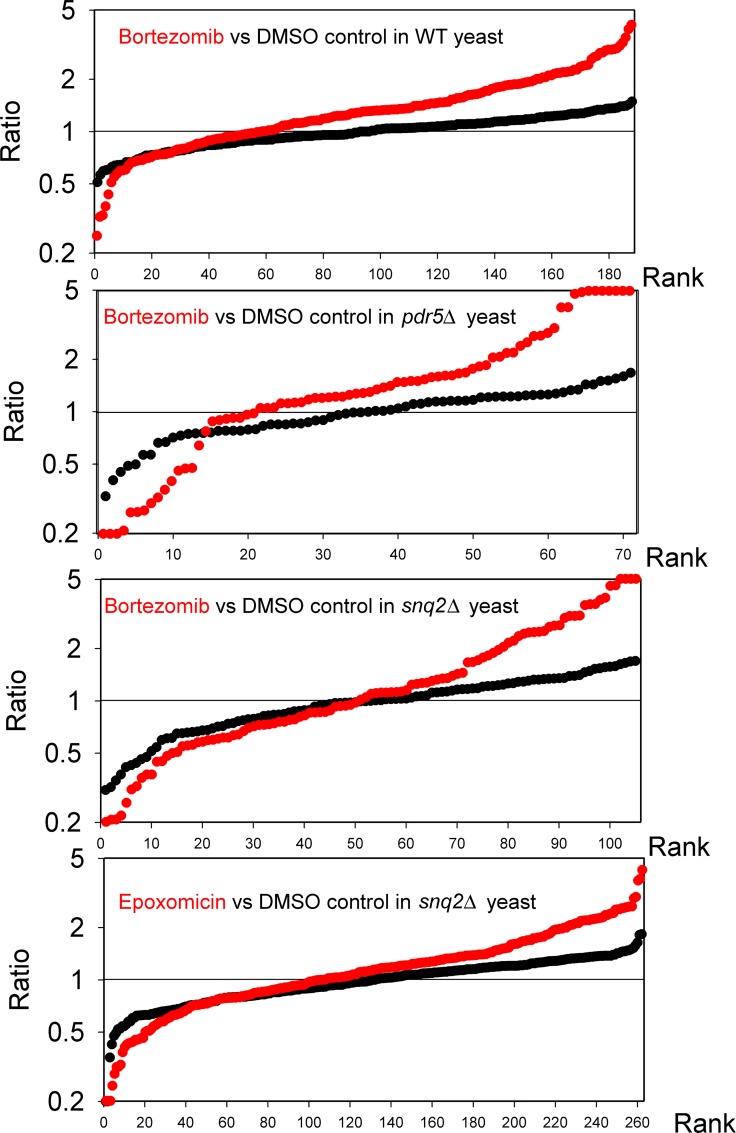
Effect of bortezomib or epoxomicin on relative levels of peptides in yeast. Wild type yeast (top panel) or yeast deleted for drug transporter genes *PDR5* (second panel) or *SNQ2* (bottom two panels) were treated with proteasome inhibitors for 1 hour and then processed for peptidomics as described in Materials and Methods. See S1 Fig for labeling scheme used for quantitative peptidomics. Wild-type yeast were treated with 1 μM bortezomib, the *pdr5*Δ and *snq2*Δ strains were treated with 10 μM bortezomib, the *snq2*Δ strain was treated with 4 μM epoxomicin and control replicates were treated with a comparable amount of drug vehicle alone (maximum 0.1% DMSO). Each replicate was compared to the average level of that peptide in the control replicates and the individual ratios sorted by rank and plotted. The y-axis represents the relative level of each replicate, comparing either drug-treated to the average control values (red) or the control replicates to the average control values (black). See S5 Table for relative peptide levels of each peptide in the various treatments.

The proteasome is known to cleave intracellular proteins into peptides, with the major active subunit (beta 5) preferring hydrophobic residues in the P1 position, and the other two active subunits (beta 1 and beta 2) cleaving at sites with acidic and basic P1 residues, respectively [25,47]. Previously, the peptides in human cell lines were found to be greatly affected by treatment of cells with proteasome inhibitors, suggesting that these peptides are proteasome products [19–21]. To test if the peptides present in yeast are produced by the proteasome, cells were incubated with bortezomib or epoxomicin for 1 hour and then processed for peptidomics. Replicates of the same strain of yeast, grown under the same conditions except for the absence of inhibitor, were similarly processed. Peptides were labeled with isotopic tags so that replicates of treated cell extracts could be directly compared to replicates of untreated cell extracts in the same LC/MS run (S1 Fig). The level of each peptide in one replicate was compared to the average intensity of the peptide in the two untreated control replicates. This analysis was performed for both of the treated samples in each LC/MS run, as well as for the two untreated control replicates; the latter provided a measure of the normal variation of the peptide between replicates. Relative levels of the peptide detected in each experiment are shown in S5 Table. To visualize all of the data, the relative level of each replicate was plotted on a rank plot; for these plots, the y-axis represents the relative level and the x-axis is the rank (Fig 3). Treatment of wild-type yeast with 1 μM bortezomib did not produce major changes in the levels of intracellular peptides (Fig 3). Subsequent analysis in which the control values were pooled for each peptide detected in three or more LC/MS runs found only one peptide to be significantly altered in the wild-type yeast treated with 1 μM bortezomib. This peptide, a fragment of the *ENO2* gene product, was elevated by 1 μM bortezomib treatment (Table 2).

**Table 2:** Peptides found in three or more LC/MS runs of yeast cell extracts.

		Control			bortez/cont in *pdr5*Δ yeast			bortez/cont in *snq2*Δ yeast			epox/cont in *snq2*Δ yeast			bortez/cont in WT yeast		
Gene	Peptide	Avg	SEM	n	Avg	SEM	p	Avg	SEM	p	Avg	SEM	p	Avg	SEM	p
ADH1	STLPEIYEK	1.00	0.08	6	1.95	0.25	**	0.93	0.21	NS	1.02	0.22	NS	ND		
APE3	SPPVDGFVGK	1.00	0.16	6	1.95	0.11	*	0.93	0.38	NS	0.85	0.39	NS	ND		
APE3	SEKLQDKIKVD	1.00	0.07	6	1.42	0.21	*	1.28	0.18	NS	0.69	0.19	NS	ND		
APE3	NIIADTKHGDPDNI	1.00	0.14	6	0.34	0.06	*	1.13	0.44	NS	0.94	0.09	NS	ND		
ENO2	AVSKVYA	1.00	0.17	8	2.58	0.17	**	2.46	1.11	*	1.27	0.26	NS	1.53	0.63	NS
ENO2	QIGTLSESIK	1.00	0.07	8	1.68	0.19	**	0.74	0.13	NS	0.99	0.27	NS	1.18	0.18	NS
ENO2	QIGTLSESIKA	1.00	0.03	8	1.41	0.02	***	1.90	0.13	***	0.97	0.19	NS	1.21	0.21	NS
ENO2	GVELADMYHS	1.00	0.13	8	1.84	0.35	*	1.76	0.77	NS	1.59	0.01	NS	2.02	0.16	**
ENO2	TAIEKKAADAL	1.00	0.05	8	1.45	0.09	**	1.32	0.06	*	1.06	0.19	NS	1.28	0.16	NS
ENO2	GHDGKVKIGLD	1.00	0.13	8	0.93	0.04	NS	1.12	0.58	NS	0.79	0.01	NS	1.30	0.15	NS
ENO2	ADLSKSKTSPY	1.00	0.09	6	0.70	0.06	NS	1.21	0.56	NS	ND			1.20	0.07	NS
ENO2	SLDGTANKSKLGA	1.00	0.06	8	0.19	0.07	***	0.20	0.01	***	1.09	0.26	NS	1.22	0.15	NS
ENO2	SLDGTANKSKLGAN	1.00	0.08	8	1.54	0.22	*	1.25	0.21	NS	0.86	0.19	NS	1.28	0.28	NS
ENO2	SLDGTANKSKLGANA	1.00	0.08	8	0.28	0.01	*	0.24	0.02	***	1.10	0.27	NS	0.90	0.19	NS
ENO2	NLDVKDQKAVDDF	1.00	0.09	6	0.47	0.01	***	0.54	0.06	***	1.00	0.10	NS	ND		
GAS1	DDVPAIEVVGNKF	1.00	0.02	6	1.09	0.17	NS	0.80	0.17	*	1.01	0.01	NS	ND		
PEP4	GKFDGIL	1.00	0.14	8	5.73	0.82	***	6.70	1.07	***	1.38	0.10	NS	1.60	0.23	NS
TDH1	YDSTHGRYKGT	1.00	0.11	6	1.10	0.04	NS	0.98	0.13	NS	ND			1.40	0.45	NS
TDH3	STGVFKEL	1.00	0.16	6	2.18	0.35	*	0.82	0.10	NS	1.08	0.07	NS	ND		
TPI1	VDIINSRN	1.00	0.13	8	0.59	0.02	NS	1.80	0.68	NS	1.83	0.80	NS	1.28	0.27	NS
TPI1	VKKPQVTVGAQ	1.00	0.06	8	2.70	0.04	***	2.06	0.41	***	1.19	0.46	NS	1.25	0.23	NS
TPI1	GAFTGENSVDQIK	1.00	0.16	8	1.31	0.24	NS	1.56	0.32	NS	2.02	0.04	*	1.33	0.17	NS
TPI1	GLAATPEDAQDIHA	1.00	0.08	8	0.34	0.13	**	0.53	0.08	*	1.44	0.07	*	1.14	0.13	NS
TPI1	ASGAFTGENSVDQIK	1.00	0.11	8	0.98	0.20	NS	0.91	0.13	NS	2.15	0.47	**	1.40	0.01	NS
TPI1	GAFTGENSVDQIKDVGAK	1.00	0.08	6	ND			2.40	0.59	**	1.23	0.14	NS	1.09	0.15	NS

Abbreviations: Avg, average; SEM, standard error of the mean; NS, not statistically significant (i.e. p>0.05); ND, not detected; and p, p-value

*, p<0.05

**, p<0.01

***, p<0.001 compared to untreated control group using Student’s t-test.

**Fig 4:**
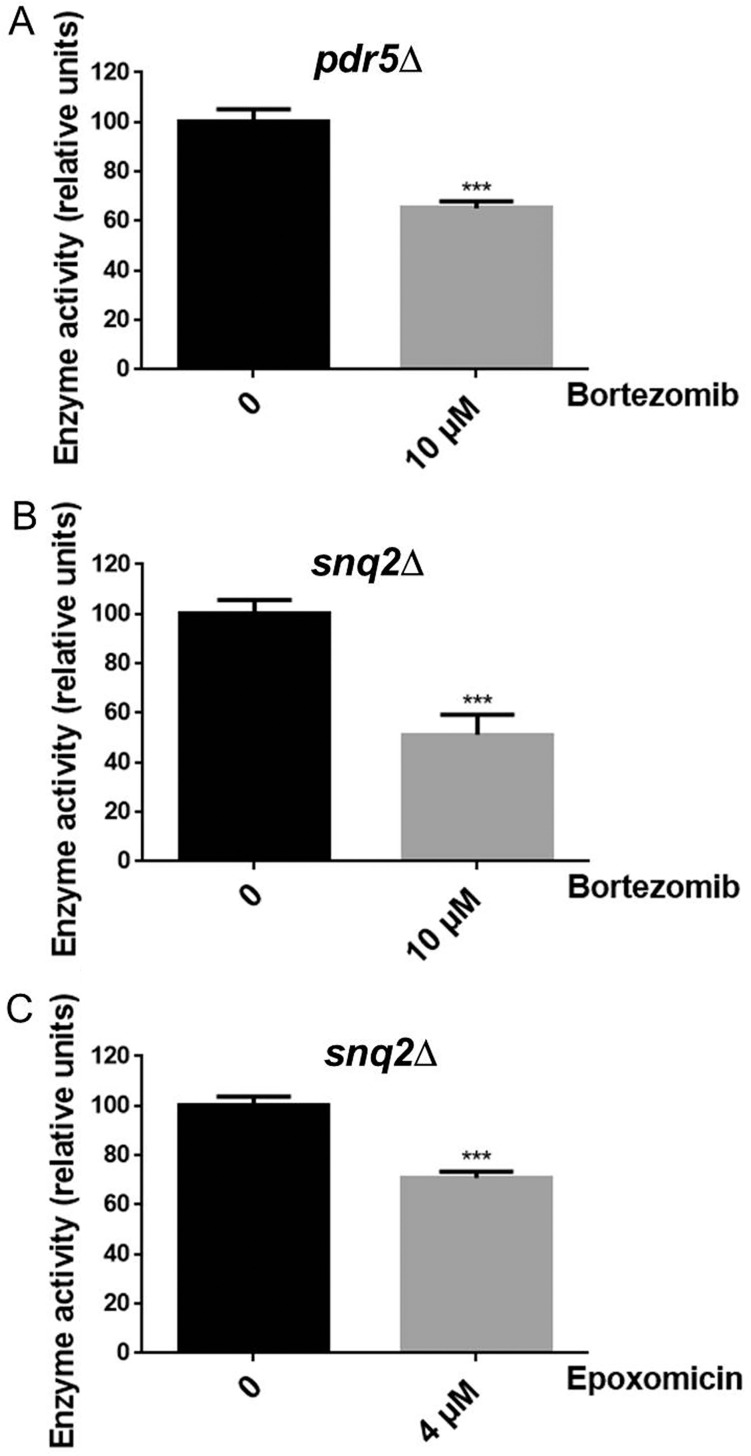
Analysis of proteasome activity in yeast. Aliquots of the *PDR5* and *SNQ2* gene deletion strains used for the peptidomics in Fig 3 were tested for proteasome activity using Suc-Leu-Leu-Val-Tyr-AMC, as described in Materials and Methods. Error bars show standard error of the mean (n = 10). ***, p<0.001 using Student’s t-test.

Because wild-type yeast contain transport proteins that actively pump bortezomib out of cells, we analyzed *PDR5* and *SNQ2* gene deletion strains; these genes encode major drug transport proteins [48]. The strains lacking the transport proteins were treated with 10 μM bortezomib for one hour, which resulted in a 30–40% reduction of chymotryptic-like proteasome activity (Fig 4). In each deletion mutant, levels of some peptides showed a dramatic change upon bortezomib treatment (Fig 3). With the *pdr5*Δ strain, approximately one half of the identified peptides showed a 2-fold change (i.e. ratio of <0.5 or >2). For the *snq2*Δ strain, approximately one third of the identified peptides showed a 2-fold change (Fig 3). Treatment of the *snq2*Δ strain with 4 μM epoxomicin for one hour did not produce major changes in the level of most peptides (Fig 3). Altogether, 25 peptides were found in three or more LC/MS runs; these peptides reflect only a subset of all peptides detected in each experiment (see S5 Table for relative levels of all peptides). The 25 commonly detected peptides were compared, pooling the control data for each experiment to increase the number of replicates (Table 2). Of these 25 peptides, 17 were significantly affected in the bortezomib-treated *pdr5*Δ strain extract, with 12 increases and 5 decreases (Table 2). Treatment of the *snq2*Δ strain with bortezomib resulted in 11 significant changes, with 6 increases and 5 decreases (Table 2). Only 3 of the peptides were altered with 4 μM epoxomicin, and all of these were elevated (Table 2). The finding that some peptides are reduced upon treatment with 10 μM bortezomib suggests that these peptides are products of proteasomal cleavage of cellular proteins. Previous studies on human cell lines found that bortezomib, epoxomicin, and other proteasome inhibitors reduced the levels of some peptides but also caused a large elevation in the levels of other peptides [19–21].

**Fig 5:**
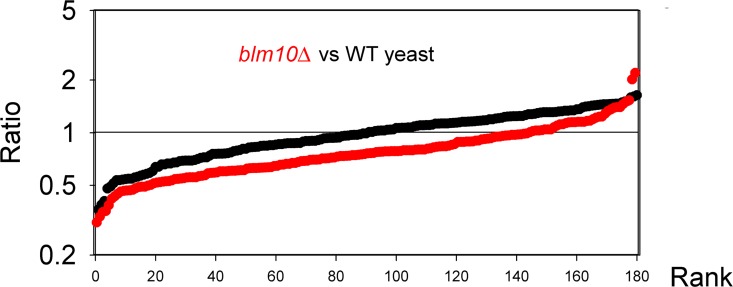
Comparison of peptide levels in wild-type and *BLM10* gene deletion yeast strains. Each replicate was plotted as described for Fig 3. See S1 Fig for labeling scheme and S5 Table for quantitative data on each individual peptide. Although there was a slight decrease in the relative level of most peptides in the *blm10*Δ strain (red) compared to the wild-type strain (black), based on the range of values for the replicates, these changes were not statistically significant.

Blm10, the yeast ortholog of the mammalian proteasome activator PA200, associates with a subpopulation of proteasomes and functions in the ubiquitin-independent degradation of protein [36,37]. In contrast, the 19S proteasome activator functions in ubiquitin-dependent protein degradation. To evaluate the relative contribution of ubiquitin-dependent and ubiquitin-independent pathways for the generation of intracellular peptides, we analyzed the peptidome of wild-type and *blm10*Δ strains (S1 Fig). All peptides found in the wild-type strain were also detected in the *blm10*Δ strain and the relative levels were generally comparable (Fig 5). None of the identified peptides shows statistically significant differences between the two groups, indicating that the absence of Blm10 does not cause a major change in the level of intracellular peptides.

**Fig 6:**
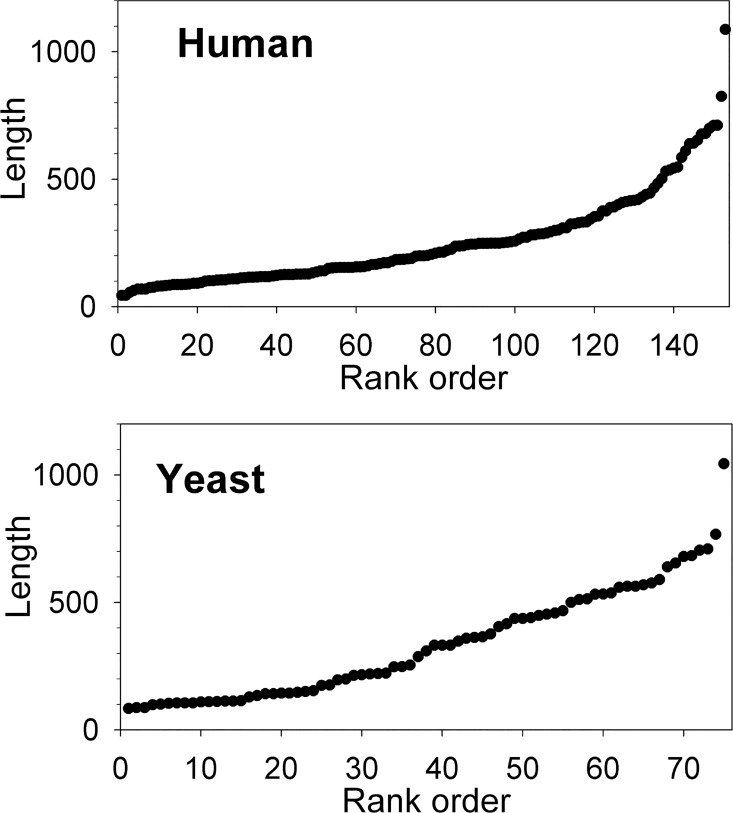
Size distribution of the 153 human proteins and 75 yeast proteins that encode the peptides detected in the peptidomic analyses. Each group of proteins was sorted by length and plotted by rank (x-axis). Units of the y-axis are amino acid residues. The average length of human proteins found in the peptidomic analysis is 253 amino acids and the median length is 199 amino acids. The average length of yeast proteins found in the peptidomic analysis is 353 amino acids and the median length is 310 amino acids.

All of the analyses described above considered the peptides themselves or the cleavage sites that generate these peptides. Another level of analysis considers the proteins from which these peptides are derived. A previous study that examined peptides associated with one of the MHC proteins, HLA-B27, found both proteasome-dependent and proteasome-independent peptides; the latter were mainly derived from small proteins [49]. Of the 153 human proteins that correspond to the 627 peptides detected in human cell lines, the average protein length is 253 amino acids and the median length is 199 amino acids (Fig 6, top); this is smaller than the average human protein size of approximately 500 amino acids [49]. Similar analysis of the 75 yeast proteins that gave rise to the peptides found in the present study show an average protein length of 353 amino acids and a median length of 310 amino acids. These values are smaller than the average protein length of 478 amino acids and median length of 384 amino acids for the 5700 yeast proteins analyzed by Arava et al [27].

The finding that the peptidome of both yeast and human cells is derived from proteins that tend to be smaller than average is contrary to the expectation that large proteins should be highly represented because they can theoretically generate more peptides and be favored in peptidomic analysis. For example, tryptic peptide fragments of equimolar amounts of five proteins were analyzed using a peptidomics approach identical to that used in the present study, and there was a strong positive correlation between the protein’s size and the number of peptides detected [43]. In the present study, a small subset of proteins is responsible for the majority of yeast peptides; 8 proteins are responsible for slightly more than half of all peptides identified (S1 Table). The average length of these 8 proteins is 385 amino acids, which is well below the average length of yeast proteins.

**Fig 7:**
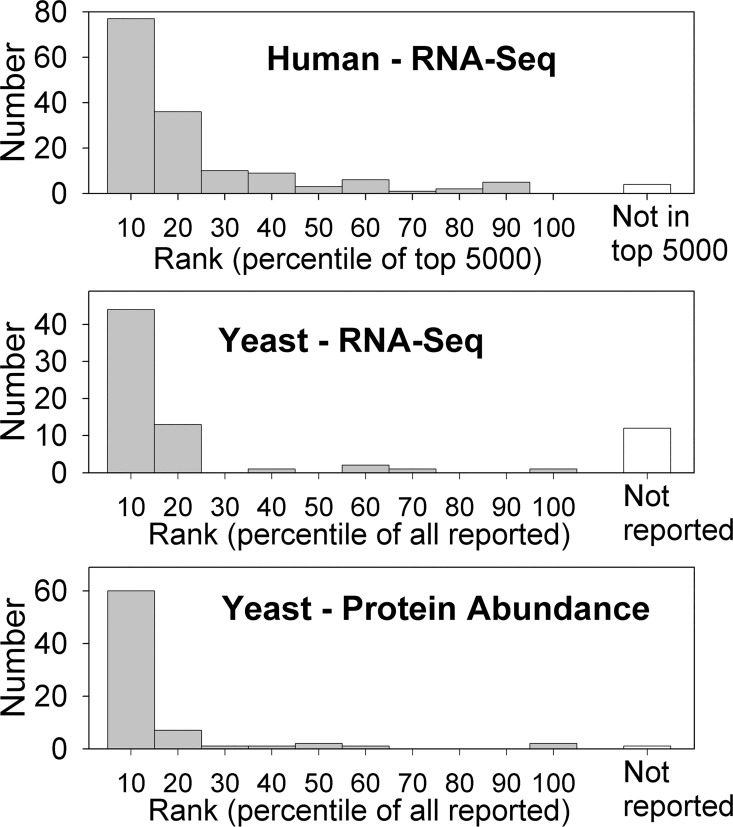
Comparison of the proteins found in the human and yeast peptidomics analyses with datasets estimating mRNA or protein abundance. RNA-Seq data for human cell lines [33] estimated mRNA levels from fragments per kilobase of transcript per million mapped reads. For each of the cell lines used for the analyses (HEK293T, SH-SY5Y, MCF7, and RPMI-8226), the 5000 entries with highest rank for any one of these cell lines were selected. These 5000 genes were divided into 10 groups of 500 genes per group, and the number of matches found in the peptidome were determined for each of these groups as well as those not within the top 5000 (of which there were 4 proteins found in the peptidome). Yeast RNA-Seq data [30] included values for 5100 genes; these were divided into 10 groups of 510. Yeast protein abundance was estimated from a label-free quantitative proteomics study by Kulak et al [51]. This study reported relative levels of 4577 proteins, and we divided these into 10 groups of 458 proteins based on abundance and the number of proteins corresponding to the peptidome is indicated for each group.

A previous analysis of the peptidome of human cell lines [17] did not find a correlation between the peptidome and protein abundance. The limitation of this analysis is that the vast majority of proteins identified in the peptidomic analysis were not detected in the proteomic study measuring protein levels [50]. To re-examine the correlation between the peptidome and protein abundance, the human peptidome was compared to RNA-seq data for the 4 cell lines from which the peptidome data was derived: HEK293T, SH-SY5Y, MCF7, and RPMI-8226 [33]. Each of the ~20,000 genes in these datasets was ordered by abundance of mRNA, and the 153 mRNAs that correspond to the peptides identified in the peptidomic analyses were selected. Of these 153 mRNAs, 77 are within the top 500 ranking of at least one of the four cell lines used for the various peptidomic studies, and all but 4 are within the top 5000 (Fig 7). Thus, many of the intracellular peptides in human cell lines are derived from proteins encoded by abundant mRNAs.

The yeast peptidome was also analyzed for a correlation with RNA and protein abundance. RNA-seq data for yeast [30] reported values for 5100 genes, and 63 of the proteins found in the peptidomic analysis were among the transcripts with values listed; 12 were not reported in the RNA-seq data (Fig 7). The 5100 genes were divided into groups of 510 based on RNA abundance. Of the 510 genes with RNA expression in the highest tenth percentile, 44 corresponded to genes detected in the peptidomics analysis. Thirteen of the genes corresponding to peptides were in the 10-20^th^ percentile of RNA expression levels, and the other 7 genes corresponding to peptides were scattered among the lower percentiles (Fig 7). Similar analysis was performed to compare the peptidome with protein abundance. Several studies have reported protein abundance; the most comprehensive of these is a recent study by Kulak et al that used MS with label-free quantification to estimate protein copy number for 4577 yeast genes [51]. These proteins/genes were divided into ~10 groups of 458 based on protein abundance, and 60 of the 75 proteins corresponding to yeast peptides were in the top 10^th^ percentile (Fig 7). Seven of the peptides were in the 10-20^th^ percentile of protein abundance, and the other 7 were distributed among the other groups (Fig 7). Similar results were found when analyses were performed for RNA or protein abundance determined from other techniques such as single molecule sequencing [31], an epitope-tagged fusion library [28], and mRNA translation profiles [27]. Taken together, these results suggest that nearly all of the peptides detected in the present study are produced from proteins that are among the top 20^th^ percentile in terms of cellular protein abundance or predicted synthetic rate. However, there are approximately one thousand proteins in this group and only a small subset was found to be represented in the peptidome. Thus, abundance alone is not sufficient to predict if peptide fragments will be detected in peptidomic studies.

**Fig 8:**
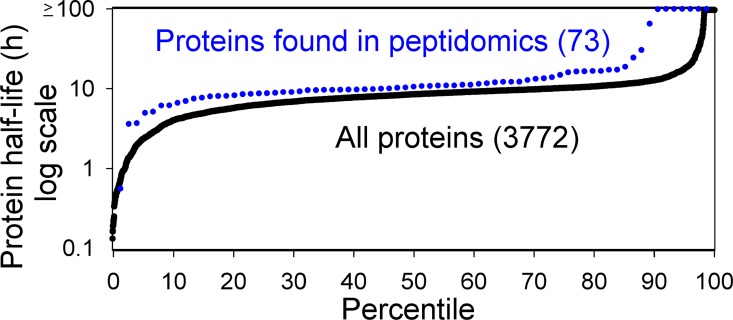
Analysis of the turnover rate of proteins found in the analysis of the yeast peptides. Protein half-life data were reported by Christiano et al [32] and were determined by growing yeast in heavy [13C6/15N2] L-lysine for many generations, then growing in light lysine for various lengths of time and measuring the ratio of heavy/light proteins using mass spectrometry-based proteomics. The half-life was capped by Christiano et al at 100 hours. Out of 75 proteins found in our peptidomics analysis, 73 corresponded to proteins detected by Christiano et al. The relative half life (log scale) for these 73 proteins and for all 3772 proteins reported by Christiano are plotted as a percentile of the half-life for each group of proteins.

In theory, the cellular peptides should arise from proteins that have high rates of turnover. The median half-life of 3772 yeast proteins examined by Christiano is 8.8 h [32]. Out of 75 proteins found in peptidomics analysis, 73 were measured in Christiano et al, and these 73 proteins have a median of 10.6 h (Fig 8). Only 1 of the 73 proteins found in peptidomics had a half-life ≤3 h, whereas 7% of all proteins had half-lives of 3 h or less [32]. Furthermore, 8% of the proteins found in peptidomics had half-lives of ≥100 h, whereas only 1% of all proteins detected by Christiano had half-lives of ≥100 h [32]. Thus, the proteins that give rise to the observed yeast peptides are more stable than average proteins, not less stable as predicted.

**Fig 9:**
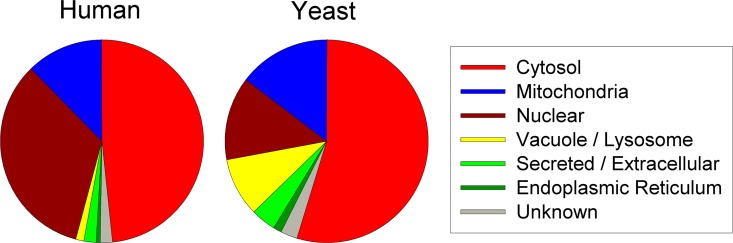
Intracellular location of proteins found in the peptidomic analysis of human and yeast cells. For each protein, the major intracellular location reported in UniProt was used. Some proteins were reported to be equally present in the cytosol as well as another compartment (such as nucleus); in these cases, the non-cytosolic compartment was selected.

Approximately 50% of the 153 human proteins identified in peptidomic studies are annotated as cytosolic, ~30% are nuclear, ~15% are mitochondrial, and ~5% from other compartments or unknown distribution (Fig 9); this is generally similar to our previous analysis performed on fewer proteins [17]. Analysis of the yeast proteins found in the present peptidomics analysis shows the majority to be derived from cytosolic proteins (Fig 9). Like the human proteins, ~15% of the yeast proteins are mitochondrial. However, only ~15% of the yeast proteins are nuclear, in contrast to the ~30% of the human proteins. A greater fraction of the yeast proteins are found in vacuoles, endoplasmic reticulum, or secretory pathway, compared to the human proteins (Fig 9).

**Fig 10:**
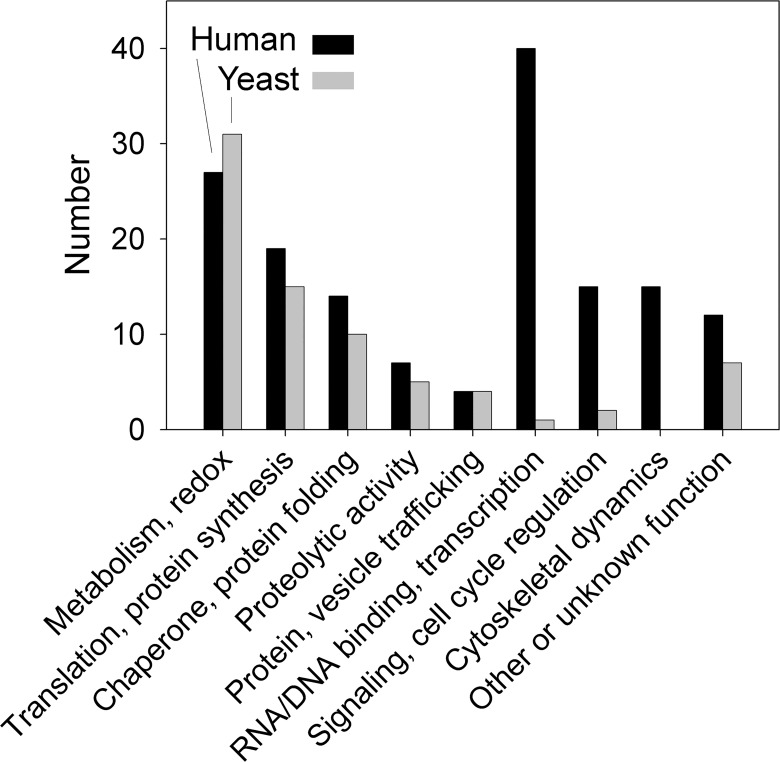
Primary function of proteins found in the peptidomic analysis of human and yeast cells. For each protein, the major function reported in UniProt was used.

Many of the proteins which contribute to the human and yeast peptidome are involved with basic cellular functions such as metabolism, maintenance of reduction/oxidation balance, translation/protein synthesis, chaperone/protein folding, protein/vesicle trafficking, and proteolysis (Fig 10). However, there are several categories of functions for the human proteins that are greatly under-represented in the yeast peptidome. Of the 153 human proteins found in the peptidome, 46 function in transcription or RNA/DNA binding and 16 function in cytoskeletal dynamics, whereas no yeast peptides were found from proteins with these functions (Fig 10). Only 2 yeast proteins that function in intracellular signaling and/or regulation of the cell cycle were found in the peptidome, whereas 21 human proteins found in peptidomic analyses function in this category (Fig 10). Taken together, these results suggest that while many of the proteins detected in the peptidome of humans and yeast have similar functions, there are some categories of human proteins that aren’t represented in the yeast peptidome.

**Fig 11:**
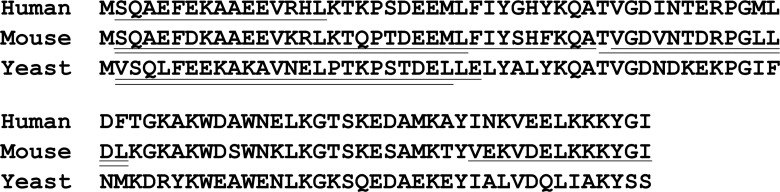
Comparison of yeast, mouse, and human acyl-CoA-binding protein. Underlined regions denote peptides found in the peptidome of yeast, mouse tissues and cell lines, and human cell lines. Double underline indicates multiple overlapping peptides found. In mouse tissues, additional N-terminal peptides were found.

To directly compare individual proteins between yeast and human, the 75 yeast proteins found in the present study were used to search for human orthologs using Blast homology searches of NCBI databases. Of these 75 proteins, 56 have a clear human ortholog, and 23 of these orthologs are found in the human and/or mouse peptidome database (Table 3). These orthologs include a number of proteins associated with ribosomes, cellular metabolic pathways, and chaperones such as 10 kDa heat shock protein. For some of these orthologs, the peptides found in the yeast peptidome arise from the same region of the protein as peptides present in the human and/or mouse peptidomes. The alignment of acyl-CoA-binding protein is shown in Fig 11, and all alignments of orthologs are included in S2 Fig. In yeast, two N-terminal fragments of acyl-Co-A-binding protein were identified, in human cells a single N-terminal fragment was identified, and in mouse tissues, a number of N-terminal peptides were identified along with a couple of internal peptides (Fig 11, underlined sequences). The overall amino acid sequence identity between yeast, mouse, and human acyl-CoA-binding protein is 37% (considering only exact matches of all three species). Within the 26 residues found in the yeast peptidome, the sequences are slightly less highly conserved, with only 31% identity. A similar comparison of all 23 orthologs in which peptides were found in yeast and at least one of the mammalian species (i.e. human and/or mouse) was performed (Table 3). The overall amino acid identity of the proteins ranged from 17% (for the SBA1 gene product) to 66% (for the SSA2 gene product), with an average of 46% (Table 3). Considering just the region corresponding to the peptides found in yeast, the amino acid sequence identity ranged from 0% (for the AHP1 gene product) to 85% (for the SSC1 gene product), with an average of 45% (Table 3). Thus, the degree of conservation of the region of each protein that was found in the yeast peptidome is comparable to the overall conservation of the proteins, and not more or less highly conserved.

**Table 3:** Comparison of proteins found in the yeast peptidome with orthologs in mammalian peptidome (human and mouse).

Yeast Gene	Human Gene	Yeast	Yeast Peptides	Yeast Protein	Conservation within peptide	Conservation entire protein
Name	Name	Protein Name	# of aa	# of aa	% aa identity	% aa identity
ACB1	DBI	Acyl-CoA-binding protein	26	87	31%	37%
AHP1	PRDX5	Peroxiredoxin type-2	17	176	0%	22%
CDC19	PKM	Pyruvate kinase 1	53	500	49%	49%
CMD1	CALM1	Calmodulin	30	147	43%	56%
ENO2	ENO2	Enolase 2	239	437	58%	61%
GPM1	PGAM2	Phosphoglycerate mutase 1	60	247	48%	51%
HSP10	HSPE1	10 kDa heat shock protein	30	106	43%	44%
PEP4	CTSD*	Vacuolar aspartyl protease	34	405	53%	36%
RPL31A	RPL31	60S ribosomal protein L31A	16	113	50%	59%
RPP1A	RPLP1	60S acidic ribosomal protein P1α	13	106	46%	51%
RPP2A	RPLP2	Ribosomal protein P2 alpha	9	106	56%	51%
RPP2B	RPLP2	Ribosomal protein P2 beta	30	110	43%	46%
RPS21A	RPS21	40S ribosomal protein S21A	41	87	56%	51%
SBA1	PTGES3	Co-chaperone protein SBA1	24	216	13%	17%
SMT3	SUMO2	Ubiquitin-like protein SMT3	25	101	44%	38%
SOD1	SOD1	Superoxide dismutase [Cu-Zn]	55	154	49%	51%
SSA2	HSPA8*	ATP binding protein	11	639	55%	66%
SSC1	HSPA9	Heat shock protein SSC1	13	654	85%	63%
STI1	STIP1	Heat shock protein STI1	25	589	8%	36%
TPI1	TPI1	Triose phosphate isomerase	67	248	55%	52%
TRX2	TXN	Thioredoxin-2	21	104	19%	38%
TSA1	PRDX1	Peroxiredoxin TSA1	46	196	59%	59%
VMA10	ATP6V1G1*	V-type proton ATPase subunit G	22	114	64%	32%

Abbreviation: aa, amino acid.

*Additional orthologs are present in the human genome.

## Discussion

The major finding of this study is that yeast cells contain peptides derived from intracellular proteins. While it is possible that some of the peptides detected in the present study and in previous studies on mammalian cell lines represent degradative products generated during protein extraction, we believe this is unlikely for several reasons. First, only a subset of proteins is represented in the peptidome, and for most proteins only a small number of peptides are detected. In contrast, non-specific degradation (such as trypsin-mediated digestion of proteins) typically produces a large number of distinct fragments from the major cellular proteins. Second, the cells (yeast in this study and mammalian cells in previous studies) were rapidly heated to 80°C, and no yeast or human proteases are known to be stable to this temperature. A number of studies have compared various methods of sample preparation for peptidomics and concluded that rapid heat inactivation is a valid method to greatly reduce postmortem protein degradation in tissues [18,23,46,52–55]. Third, the finding that the vast majority of intracellular peptides detected in mammalian cell lines are greatly reduced by short-term treatment of cells with proteasome inhibitors such as epoxomicin and MG132 strongly suggests that these are naturally occurring products of proteasome activity, and not artifactually-generated peptides [19,21]. Finally, the finding that the yeast peptidome is remarkably similar to the peptidome of human and mouse cells suggests that the generation and/or stability of the intracellular peptides is conserved. Approximately one third of the yeast proteins that give rise to peptides detected in the present study have human/mouse orthologs that give rise to peptides found in previous studies on human and mouse cell lines and mouse tissues. In some cases, the yeast and mammalian peptides arise from the same general region of the proteins, but these regions do not appear to be more highly conserved than the parts of the protein that were not found in the peptidome. This contrasts with proteins encoding neuropeptide precursors, in which the regions corresponding to the bioactive peptides are more highly conserved than the other regions of the proteins [56]. The proteasome is known to cleave proteins into peptides, although the dogma in the field is that these peptides are rapidly degraded by intracellular aminopeptidases [57]. However, there is no direct evidence to support this dogma, and studies on the stability of peptides within cells have found that a subset appear to be more stable than others [9,10]. Because proteasome inhibitors such as epoxomicin alter the levels of nearly all of the peptides identified in human cells, it is likely that these peptides are proteasome products. The small number of yeast peptides that were significantly affected by treatment with proteasome inhibitors is consistent with the modest proteasome inhibition (30–40%) achieved in the present study. Studies of HEK293T cells with 50 nM bortezomib for 1 hour caused a ~70% inhibition of proteasome activity and altered the level of most peptides, whereas a ten-fold lower dose of bortezomib resulted in 15% inhibition and produced few changes in peptide levels [20,21]. Despite using μM concentrations of inhibitors and yeast strains that lack several of the major drug efflux pumps, only modest inhibition of proteasome activity was achieved, likely due to the efficiency of the remaining drug efflux pumps in the organism. Interestingly, the paradoxical increase in levels of some peptides upon treatment of human cells with bortezomib [20] was also observed in yeast (Fig 3, Table 2). Several mechanisms have been proposed for this effect, including bortezomib-mediated inhibition of downstream peptidases or changes in the proteasome structure [20,22]. The finding that yeast peptides are also elevated by treatment with bortezomib suggests that the mechanism for this paradoxical effect is common to yeast and human cells.

The vast majority of yeast peptides arise from proteins that are among the top 20^th^ percentile in terms of abundance (Fig 7), based on a variety of large-scale approaches to measure levels of protein, RNA, transcription, or translation [27,28,30–32,51]. A similar comparison of the human cell line peptidome with RNA-seq data for those cell lines [33] also shows a strong correlation of peptides with mRNA abundance (Fig 7). Although peptides in both yeast and human cells are largely derived from abundant proteins and/or mRNAs, abundance alone is not sufficient to predict whether peptides will be detected; only a small subset of the most abundant proteins were detected in the peptidome. Furthermore, most proteins were represented by only one or two peptides, with N- and C-terminal fragments highly represented in both the yeast and human cell line peptidomes. In theory, <1% of peptides should be derived from the N- or C-termini of a protein, based on an average protein length of 300–400 amino acids and average peptide length of 15 amino acids. Proteomic studies that digest proteins with trypsin typically detect mostly internal peptides and only a small fraction of N- or C-terminal peptides, which indicates that mass spectrometry does not favor N- or C-terminal fragments [43]. One possibility to account for the high fraction of N- and C-terminal peptides is that they are selectively produced within the cell. Another possibility is that N- and C-terminal peptides may be more stable than other fragments. However, the amino acid composition of the peptides detected in our analysis is comparable to the amino acid composition of intracellular and nuclear yeast proteins, and is not enriched for amino acids that are resistant to proteases. Alternatively, the peptides could be protected from proteases by binding to intracellular proteins. This latter idea is consistent with the proposal that intracellular peptides may have specific functions within cells that involve binding to other proteins [15,16].

In higher eukaryotes, endogenous intracellular peptides have been shown to regulate signal transduction [58,59], intracellular calcium levels [59], and glucose uptake [60]. These studies treated cells with synthetic peptides which corresponded to peptides identified in peptidomics analyses of cells or tissues. A study in *C*. *elegans* found evidence that peptides produced by proteolysis of mitochondrial proteins were released into the cytosol where they interacted with transcription factors to regulate the mitochondrial unfolded protein response [61]. Endogenous peptides encoded by mRNAs with short open reading frames were shown to affect transcription in *Drosophila* [62]. These peptides induce proteasome-mediated processing of a transcription repressor into a shorter activator [63]. Many additional studies have used synthetic peptides to perturb protein folding or protein-protein interactions; for review see [11,12,64–66]. Thus, peptides can clearly have a number of activities within cells.

The proteasome is commonly known to function in the degradation of unneeded or damaged proteins. However, it is equally valid to consider the eukaryotic proteasome as a producer of intracellular peptides. The catalytic core of the eukaryotic proteasome is structurally related to the archaebacterial proteasome, which contains seven identical catalytically active subunits [25]. Evolution added complexity to the comparatively simple prokaryotic proteasome—in eukaryotes there are seven distinct beta subunits, only three of which are catalytically active. This feature is conserved from yeast to humans, suggesting that the proteasome may have evolved from a purely degradative function in prokaryotes to a peptide-generating function in eukaryotes. In mammals, a small fraction of the peptides produced by the proteasome are displayed on cells in complex with MHC proteins and play a central role in the recognition of foreign molecules. However, this function is only needed in higher eukaryotes with a functional immune system (i.e. jawed vertebrates) [6]. The finding of the present study that peptides are present in yeast, and that some of the peptides arise from orthologs of proteins found to produce intracellular peptides in human cells, supports the idea that the production of intracellular peptides is an important biological process.

## Supporting Information

S1 Appendix Manual interpretation of Mascot results: Example of a peptide that matches all of the criteria. Every peptide tentatively identified by Mascot is manually reviewed and must match a number of criteria. These criteria are described this Appendix along with an example of a peptide that is representative of a “weak” Mascot score, with discussion of the decision process involved in accepting the identification.
(PDF)

S2 Appendix Manual interpretation of Mascot results: Example of a peptide that does not match the criteria. This Appendix shows an example of a peptide that has a “weak” Mascot score and which was rejected because it failed to meet the criteria.
(PDF)

S1 Fig Schematic diagram of TMAB labeling strategy for each LC/MS run on yeast peptides. Four of the experiments started with two independent cultures each of wild-type (WT) or *pdr5*Δ or *snq2*Δ mutant yeast strains (top four panels). After growth to early log phase, each culture was split into two equal volumes; one of which was treated for one hour with the indicated proteasome inhibitor (bortezomib or epoxomicin, dissolved in DMSO), the other treated with a comparable amount of DMSO (maximum 0.1%). Thus, each experiment included two independent biological replicates for each control and treated strain. These four cultures were then processed for peptidomics as described in Materials and Methods. The labeling strategy for comparison of WT and *blm10*Δ strain yeast strains is shown in the lower panel. In all experiments, peptides were labeled with TMAB-NHS isotopic labels as indicated.
(PDF)

S2 Fig Alignments of proteins that are conserved between human and yeast. The regions of the proteins that correspond to peptides found in the peptidome are indicated by underline. In some cases, multiple peptides were found and are indicated by a double underline. If more than 2 peptides found for a particular region of the protein, the additional peptides are not indicated.
(PDF)

S1 Table Yeast peptides (one row per peptide). Information includes peptide sequence, mass, flanking residues, and name of gene and protein.
(XLSX)

S2 Table Yeast proteins that give rise to the peptides reported in S1 Table (one row per protein/gene). Information includes protein length, intracellular location, function, and gene name.
(XLSX)

S3 Table Human peptides (one row per peptide). Information includes peptide sequence, mass, flanking residues, and name of gene and protein.
(XLSX)

S4 Table Human proteins that give rise to the peptides reported in S3 Table (one row per protein/gene). Information includes protein length, intracellular location, function, and gene name.
(XLSX)

S5 Table Quantitation of yeast peptides in each LC/MS run. Information includes peptide sequence, gene name, and relative levels in each replicate for the experiments listed in S1 Fig.
(XLSX)
