# Supplementary material for: Analysis of the Yeast Peptidome and Comparison with the Human Peptidome
Source: PLoS One. 2016 Sep 29;11(9):e0163312. doi: 10.1371/journal.pone.0163312 (PMC5042401; doi:10.1371/journal.pone.0163312)
Supplement: S1 Appendix — Every peptide tentatively identified by Mascot is manually reviewed and must match a number of criteria. These criteria are described this Appendix along with an example of a peptide that is representative of a “weak” Mascot score, with discussion of the decision process involved in accepting the identification. (PDF) [file pone.0163312.s001.pdf]

Appendix 1. Manual interpretation of Mascot results: Example of a peptide that matches all of the criteria

Dasgupta et al, 2016

Overview: Every peptide tentatively identified by Mascot is manually reviewed and must match a number of criteria. These criteria are listed below. While the Mascot score is a factor and those with scores above 50 are very likely to be correct, there is no rigid cut-off in score because some high-scoring peptides are clearly false positives (based on the criteria) while some peptides with moderate scores show convincing MS/MS spectra and match the criteria—these would be false negatives if the score was the only factor considered.

This Appendix shows an example of a peptide that is representative of a “weak” Mascot score. Most peptides indicated in Table S1 had much higher scores. Rather than show a peptide with a high score, it is more appropriate to show a peptide with a borderline score and to walk through the decision process involved in either accepting or rejecting the identification.

Criteria: (reprinted with minor modification from “Quantitative peptidomics of mice lacking peptide-processing enzymes.” Wardman J, Fricker LD. Methods Mol Biol. 2011; 768:307-23).

1. The isotopic form of TMAB matched by Mascot is the correct one based on analysis of the peak set. While this may seem obvious, Mascot does not consider the peak set and know which of the individual peaks correspond to the appropriate form. If using the five isotopic forms of TMAB, there is a 1 in 5 chance that a false positive labeled with one tag is correct. If two tags, there is a 1 in 25 chance that a false positive has the correct tags. Therefore, a correlation of the isotopic TMAB form in the observed peak set with the predicted Mascot match is a simple and necessary step that excludes the majority of false positives.

2. The number of tags incorporated into the peptide matches the number of free amines (N-terminus and side chains of Lys), with allowance for exceptions where labeling is incomplete. If multiple tags, all should be the same isotopic form on a particular peptide (i.e. all D0-TMAB, or D9-TMAB, and not one D0-TMAB and one D9-TMAB on the same form of a peptide).

3. The Mascot score is either the top score of all potential peptides, or the other peptides with comparable scores can be excluded by the other criteria, leaving only one peptide that matches all criteria.

4. The majority (>80%) of the major MS/MS fragment ions match predicted a, b, or y ions, or precursor ions with loss of trimethylamine.

5. The mass accuracy of the peptide and the fragment ions are within the accepted specification for the q-TOF instrument used for the analysis.

6. A minimum of 5 fragment ions match b or y ions. For small peptides, this can be a problem.

7. The charge state should be reasonable based on the peptide sequence. One positive charge for each TMAB group and for each Arg residue. If His residues are present, the peptide is usually detected in two different charge states.

These criteria are further explained in the following example for the peptide AALAPKIGPL from the protein 60S ribosomal protein L12A.

The Mascot Summary page (below) shows the 38th through the 42nd ranked "hits" from a search of one sample (named "snqdel2_Epox") against the yeast open reading frame database (6717 sequences; 3020751 residues), allowing for the D0-TMAB tags (named GIST in Mascot) on either the N-terminus or the Lys side chain and also allowing for the following variable modifications: Acetyl (N-term), Oxidation (M), and Cyano (C). Additional searches (not shown) were done with all other TMAB tags that are available on Mascot (i.e. the D3, D6, and D9 tags). Other key search parameters were: Peptide Mass Tolerance ± 0.01 Da; Fragment Mass Tolerance ± 0.2 Da; and Instrument type ESI-QUAD-TOF. The vast majority of the proteins with better Mascot scores had at least one peptide that passed the above criteria. For the peptides shown in the Mascot Summary below, only the peptide AALAPKIGPL from the protein 60S ribosomal protein L12A (i.e. the 38th ranked result) matched the criteria, as described below—**the others failed to meet one or more criteria**. See Appendix 2 for an example of a peptide that did not meet the criteria. Note that the peptide mass difference for the AALAPKIGPL peptide is within 0.0008 and 0.0003 Da.

38. YEL054C **Mass:** 17812 **Score:** 23 **Matches:** 2(0) **Sequences:** 1(0)
RPL12A SGDID:S000000780, Chr V from 53218-52721, reverse complement, Verified ORF, ""Protein component of the large (60S) ribosomal subunit, nearly identical to Rpl12Bp; rpl.

Query	Observed	Mr(expt)	Mr(calc)	Delta	Miss	Score	Expect	Rank	Unique	Peptide
190	539.3555	1076.6965	1076.6957	0.0008	0	(16)	1.3	1	U	S.AALAPKIGPL.G
311	602.9051	1203.7957	1203.7955	0.0003	0	22	0.23	1	U	S.AALAPKIGPL.G

Proteins matching the same set of peptides:
YDR418W **Mass:** 17812 **Score:** 23 **Matches:** 2(0) **Sequences:** 1(0)
RPL12B SGDID:S000002826, Chr IV from 1301609-1302106, Verified ORF, ""Protein component of the large (60S) ribosomal subunit, nearly identical to Rpl12Ap; rpl12a rpl12b doub.

39. YJL088W **Mass:** 37821 **Score:** 21 **Matches:** 2(0) **Sequences:** 2(0)
ARG3 SGDID:S000003624, Chr X from 268793-269809, Verified ORF, ""Ornithine carbamoyltransferase (carbamoylphosphate:L-ornithine carbamoyltransferase), catalyzes the sixth st

Query	Observed	Mr(expt)	Mr(calc)	Delta	Miss	Score	Expect	Rank	Unique	Peptide
531	700.3650	1398.7155	1398.7103	0.0052	0	21	5.8	1	U	M.STTASTPSSLRHL.I
671	765.4157	1528.8169	1528.8097	0.0072	0	2	5.9e+002	5	U	F.NISLDEVNKGINS.K

40. YLR430W **Mass:** 252339 **Score:** 19 **Matches:** 10(0) **Sequences:** 10(0)
SEN1 SGDID:S000004422, Chr XII from 993431-1000126, Verified ORF, ""Presumed helicase required for RNA polymerase II transcription termination and processing of RNAs; homolo

Query	Observed	Mr(expt)	Mr(calc)	Delta	Miss	Score	Expect	Rank	Unique	Peptide
38	446.7700	891.5255	891.5290	-0.0035	0	1	1.5e+002	9	U	A.KRFSNKL.T
100	492.3177	982.6208	982.6175	0.0033	0	9	43	1	U	P.LIQDIIIR.S
137	510.2790	1018.5435	1018.5481	-0.0045	0	13	46	6	U	L.ESCVRLIIS.T
435	656.8834	1311.7522	1311.7472	0.0050	0	19	7.7	1	U	R.LKDGPGMDIL.N
1264	574.3307	2293.2939	2293.3014	-0.0075	0	1	4.6e+002	3	U	S.LKSIPKACLMNSATALLRAV.L
1336	487.2825	2431.3763	2431.3758	0.0005	0	9	80	2	U	D.KKKNNKKAESPSTSSGTKKKS.S
1435	671.3445	2681.3488	2681.3404	0.0084	0	1	7.2e+002	10	U	A.HILAVSDIICSTLSGSAHDVLATMGI.K
1497	728.1312	2908.4955	2908.5005	-0.0049	0	0	1.1e+003	4	U	K.KSSIFGGMSVPSAVVPKTFPDVDSNKKA.A
1569	681.9425	3404.6761	3404.6666	0.0096	0	9	1.2e+002	1	U	T.LRSEIYCVKVMQMTTIEREYSTLEGLE.Y
1609	822.0036	4104.9816	4104.9779	0.0038	0	10	96	4	U	L.SKILADEDASQGFWSCIFSSDKHLYQAATNILYNT.F

41. YOR070C **Score:** 18 **Matches:** 1(0) **Sequences:** 1(0)
GYP1 SGDID:S000005596, Chr XV from 457822-455909, reverse complement, Verified ORF, "Cis-golgi GTPase-activating protein (GAP) for the Rab family members Yptlp (in vivo) and

Query	Observed	Mr(expt)	Mr(calc)	Delta	Miss	Score	Expect	Rank	Unique	Peptide
38	446.7700	891.5255	891.5252	0.0004	0	18	2.9	1	U	V.CAAFLIK.W

42. YLR297W **Score:** 18 **Matches:** 1(0) **Sequences:** 1(0)
YLR297W SGDID:S000004288, Chr XII from 724046-724435, Uncharacterized ORF, "Putative protein of unknown function; green fluorescent protein (GFP)-fusion protein localizes to

Query	Observed	Mr(expt)	Mr(calc)	Delta	Miss	Score	Expect	Rank	Unique	Peptide
38	446.7700	891.5255	891.5178	0.0077	0	18	3	2	U	M.IFISLRSG.S

The peptide AALAPKIGPL from the protein 60S ribosomal protein L12A was tentatively identified from two ions: m/z 602.9 (representing the peptide with 2 D0 isotopic tags—one on the N-terminus and the other on the internal Lys) and 539.4 (representing the peptide with a D0 tag only on the Lys). The Mascot scores, 22 and 16 respectively, are not very high and would

likely be rejected by high throughput approaches. Most peptides with scores in this range did not match all of the criteria listed above.

The first step in manual evaluation of the data is to look at the MS spectra to see if the ion with m/z = 602.9 that eluted at 21.9' is really a 2+ ion with 2 D0-TMAB tags, and the ion with m/z = 539.4 is really a 2+ ion with 1 D0-TMAB tag.

The MS spectra of the m/z 602.9 peak group (shown below) shows that this ion is 2+. From the spacing between peaks, it is clear that they each are different by 6 Da, meaning that they were labeled with 2 TMAB tags. Finally, it is clear that the 602.9 peak represents the peptide labeled with D0. Similar analysis of the 539.4 peak eluting at 21.6' revealed it to be a 2+ ion with 1 D0-TMAB tag (not shown). **These results match Criteria 1, 2, and 7. The peptide is labeled with the number of tags expected (two tags, one on the N-term and the other on the Lys), both of the tags are the same (D0, in this example), and the charge state is correct (2+, reflecting the two positively charged TMAB tags and no other positive charge such as Arg or His residues). The vast majority of false positives do not match the observed charge state and isotopic tags (number of tags and isotopic form).**

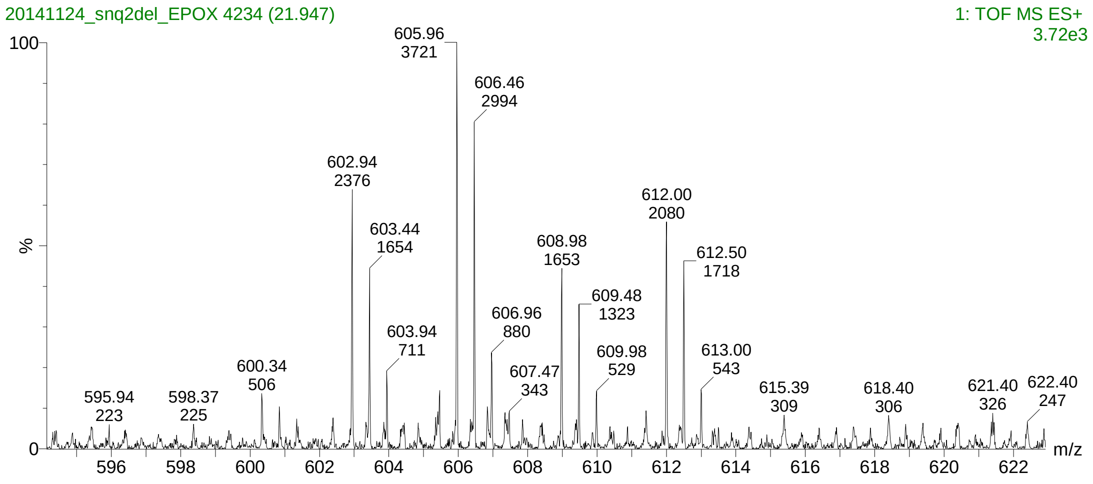

The second step in the manual verification is to examine the detailed Mascot Search Results that indicate the top scoring peptides for the MS/MS spectra. These results (shown below) indicate that the peptide AALAPKIGPL is the top scoring peptide, much higher than the next-ranked peptide on the list. **This matches criterion #3.**

All matches to this query

Score	Mr(calc)	Delta	Sequence
21.7	1203.7955	0.0003	AALAPKIGPL
3.5	1203.7955	0.0003	GGLLKKPPLL
3.5	1203.7955	0.0003	GGLLKKPPLL
1.5	1203.7954	0.0003	IKYSKIKA
1.5	1203.7954	0.0003	IKYSKIKA
1.5	1203.7954	0.0003	KISYKLAK
1.5	1203.7954	0.0003	KISYKLAK
1.5	1203.7954	0.0003	LSKKYLAK
1.5	1203.7954	0.0003	LSKKYLAK

To check the remaining criteria, it was necessary to look at the MS/MS spectra. The Mascot interpretation of the MS/MS data is shown in the summary report, although this does **not** always match the actual MS/MS spectra. In this example, the Mascot summary for the 602 m/z ion (shown below) shows a major peak with m/z~608 that doesn't match expected fragments. All other major fragments match predicted fragments.

Peptide View

MS/MS Fragmentation of **AALAPKIGPL**
Found in **YEL054C** in **Yeast_ORF**, RPL12A SGDID:S000000780, Chr V from 53218-52721, reverse complement, Verified ORF, ""Protein component of the large (60S) ribosomal subunit, nearly identical to Rpl12Bp; rpl12a rpl12b double mutant exhibits slow growth and slow translation; has simil

Match to Query 311: 1203.795708 from(602.905130,2+) intensity(460057.0000) scans(5747-5748) rawscans(fn4ix707:fn4ix708) rtinseconds(1317.467-1317.692) index(1433)
Title: 1434: Sum of 2 scans in range 5747 (rt=1317.47, f=4, i=707) to 5748 (rt=1317.69, f=4, i=708) [C:\Users\Administrador\Desktop\Synapt_24_11_2014\20141124_snq2del_EPOX.raw]
Data file 20141124_snq2del_EPOX.mgf

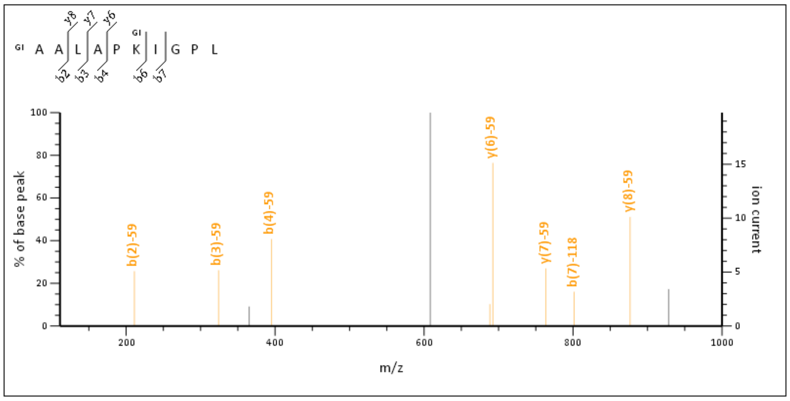

111.11 to 1000

Label all possible matches   Label matches used for scoring

Monoisotopic mass of neutral peptide Mr(calc): 1203.7955
Variable modifications:
N-term : GIST-Quat (N-term), with neutral loss 59.0735
K6 : GIST-Quat (K), with neutral loss 59.0735
Ions Score: 22 **Expect:** 0.23
Matches : 8/54 fragment ions using 14 most intense peaks (help)

The 608 peak appears to be a 2+ ion, based on the MS/MS data (shown below).

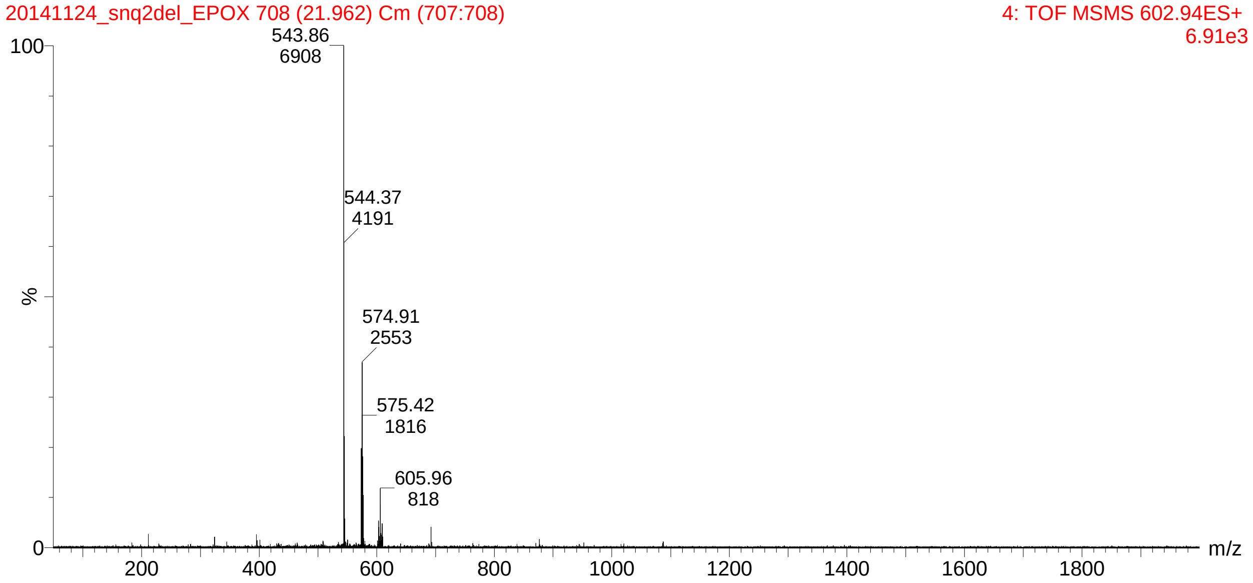

Top: the full scan of the MS/MS spectra for the 602 m/z ion that eluted at 21.9'. Bottom: zoom of the region from m/z 530 to 625.

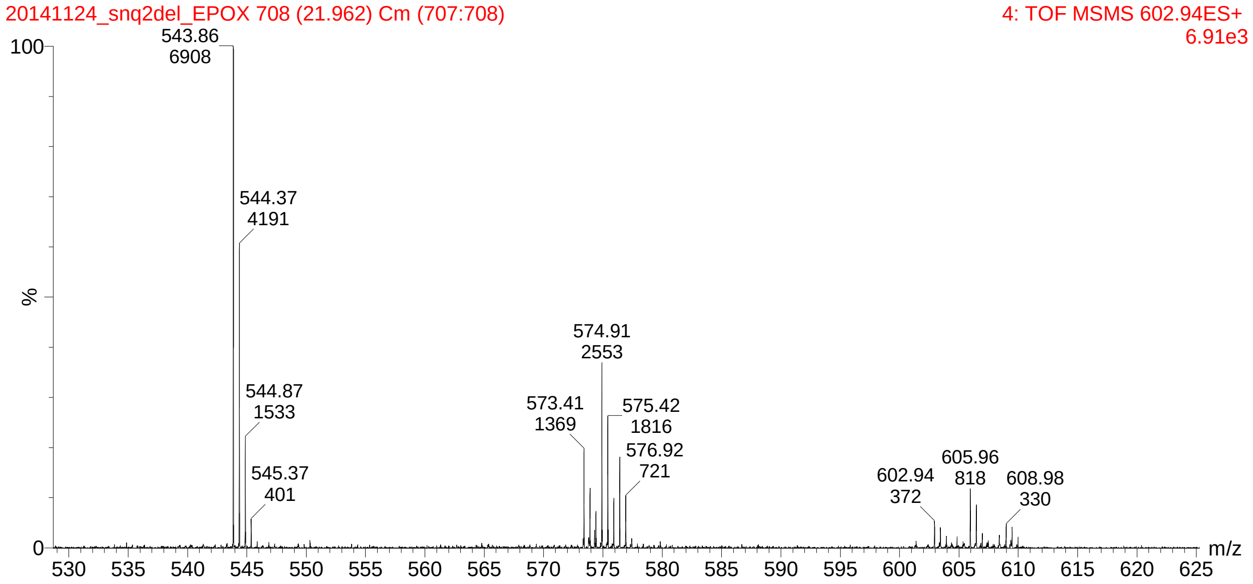

The 602-609 peak group represents the precursor peptide with 2 trimethylamino (TMA) moieties (this is similar to the MS spectra shown on page 3 of this Appendix). The peak group at m/z 573-577 represents the peptide with 1 TMA remaining, and the peak at 543.86 represents the peptide after loss of both TMA moieties. The TMA group is labile and is cleaved from the butyrate group upon collision-induced dissociation, thereby removing the isotopic difference between the tags while leaving behind a butyrate group that needs to be considered in the interpretation of the fragment ions.

The MS/MS spectra of the m/z 602 ion over the range 50-525 is shown below.

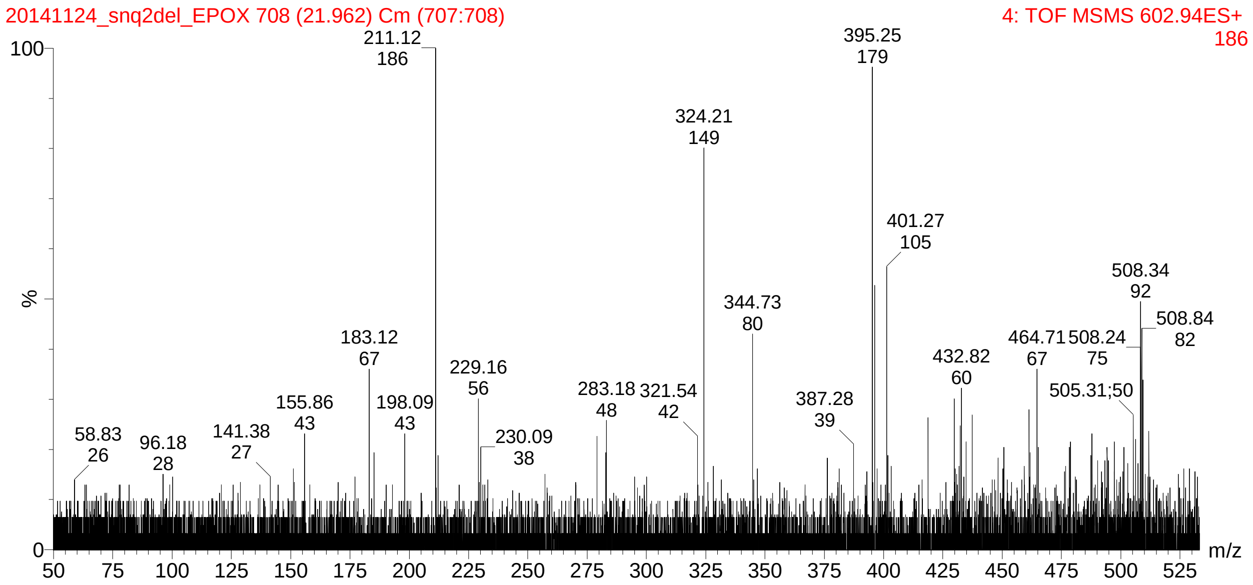

The MS/MS spectra of the m/z 602 ion over the range 620-1120 is shown below.

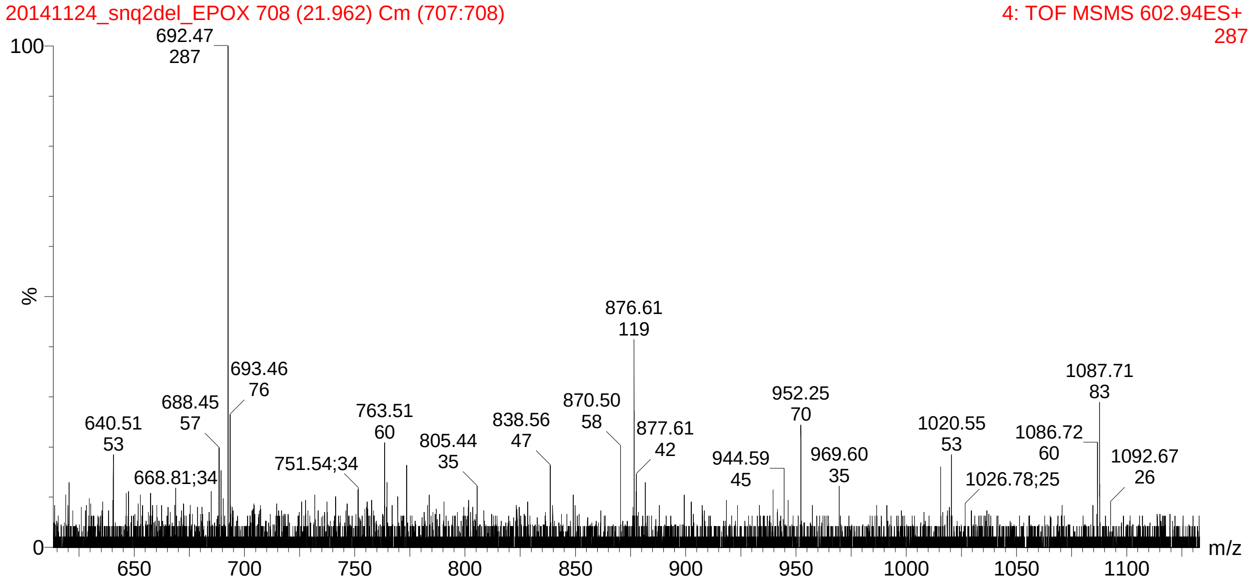

The major fragments observed in the MS/MS spectra of the m/z 602 ion fit with those predicted, which are listed in the Mascot Search Results table shown below. Note that the fragments resulting from breakage of the Ala-Pro bond are strong in the MS/MS spectra (395 b ion and 692 y ion). Xaa-Pro bonds are especially sensitive to collision-induced dissociation, and therefore the observation of strong signals that match the predicted fragments is further support. Also note the strong signal for b2 ion (211) and also a2 ion (183); both b2 and a2 ions are often strong.

#	b	b++	b*	b*++	Seq.	y	y++	y*	y*++	#
1	140.0706	70.5389			A					10
2	***211.1077***	106.0575			A	947.5924	474.2999	930.5659	465.7866	9
3	***324.1918***	162.5995			L	**876.5553**	438.7813	859.5288	430.2680	8
4	***395.2289***	198.1181			A	**763.4713**	382.2393	746.4447	373.7260	7
5	492.2817	246.6445			P	**692.4341**	346.7207	675.4076	338.2074	6
6	***688.4028***	344.7051	671.3763	336.1918	K	595.3814	298.1943	578.3548	289.6811	5
7	***801.4869***	401.2471	784.4604	392.7338	I	399.2602	200.1337			4
8	858.5084	429.7578	841.4818	421.2445	G	286.1761	143.5917			3
9	955.5611	478.2842	938.5346	469.7709	P	229.1547	115.0810			2
10					L	132.1019	66.5546			1

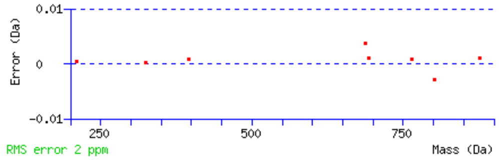

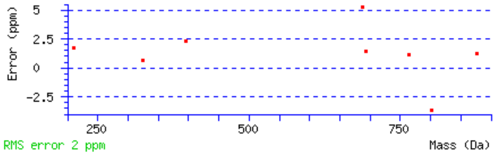

NCBI **BLAST** search of AALAPKIGPL
(Parameters: blastp, nr protein database, expect=20000, no filter, PAM30)
Other BLAST web gateways

In comparing the spectra and table shown above, it is clear that the observed fragments match b and y series ions, without loss of water (b*, y*). False positives tend to show random matches to masses on the list, including many with loss of 18 Da.

The observed fragments are within a few ppm (0.01 Da) of the predicted masses, as evident in the above figure. The window of the search parameter for Fragment Mass Tolerance was ± 0.2 Da, and false positives tend to show more variation in error than 0.01 Da.

Therefore, based on consideration of the MS/MS spectra, criteria 4, 5 and 6 are met for the m/z 602 ion.

Similar analysis was performed for the 539 m/z ion found in the above Mascot search (which considered peptides modified with D0-TMAB), and for the 542 ion found in another Mascot search which considered peptides modified with D6-TMAB. These were tentatively identified as the same AALAPKIGPL peptide tagged with 1 TMAB on the Lys residue. The MS/MS of fragmentation of the 539 m/z and the 542 m/z are shown below. Note that the fragmentation pattern is nearly identical, reflecting the loss of the TMA moiety and hence the isotopic mass difference during CID fragmentation.

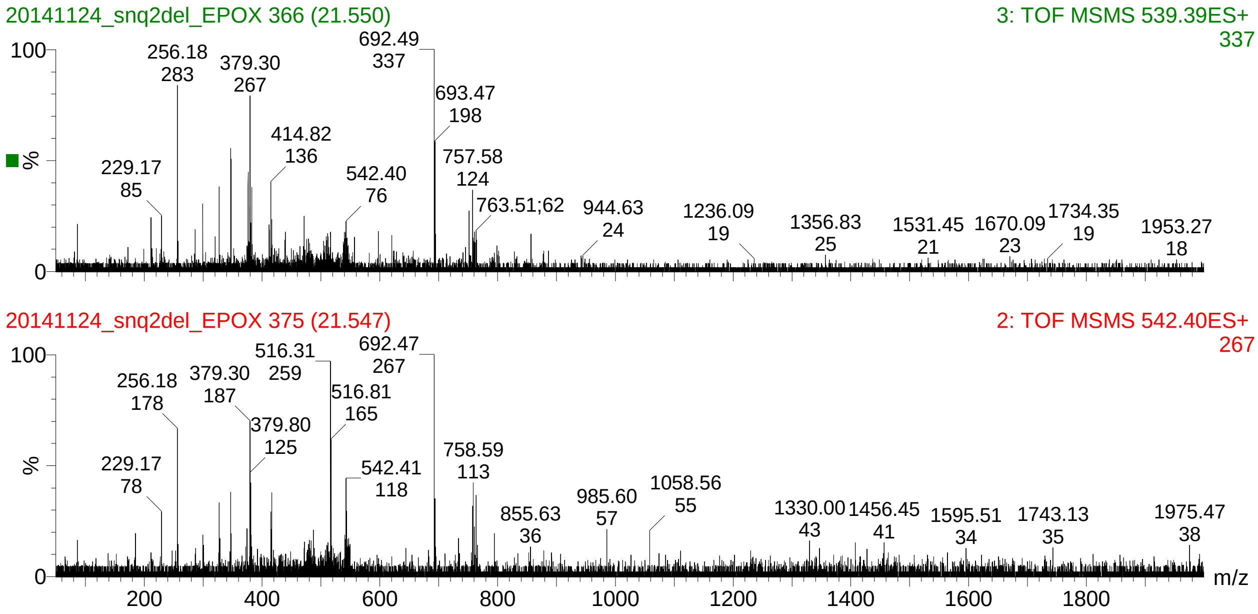

The following spectra are a zoom of the above, showing the lower mass range.

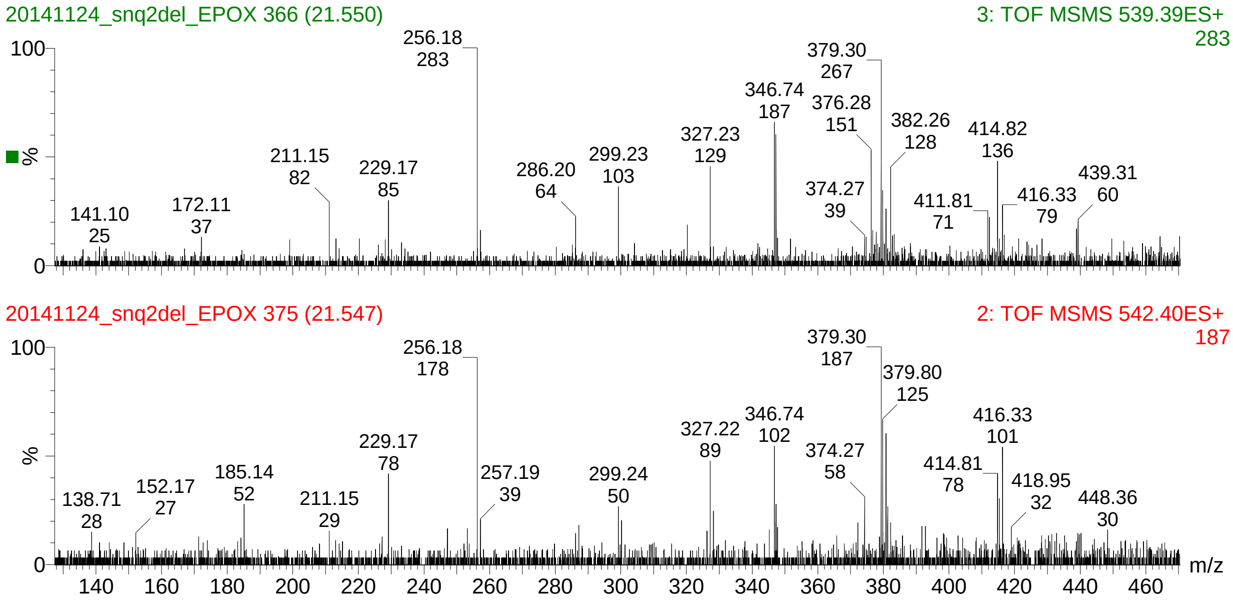

The following spectra are a zoom showing the higher mass range.

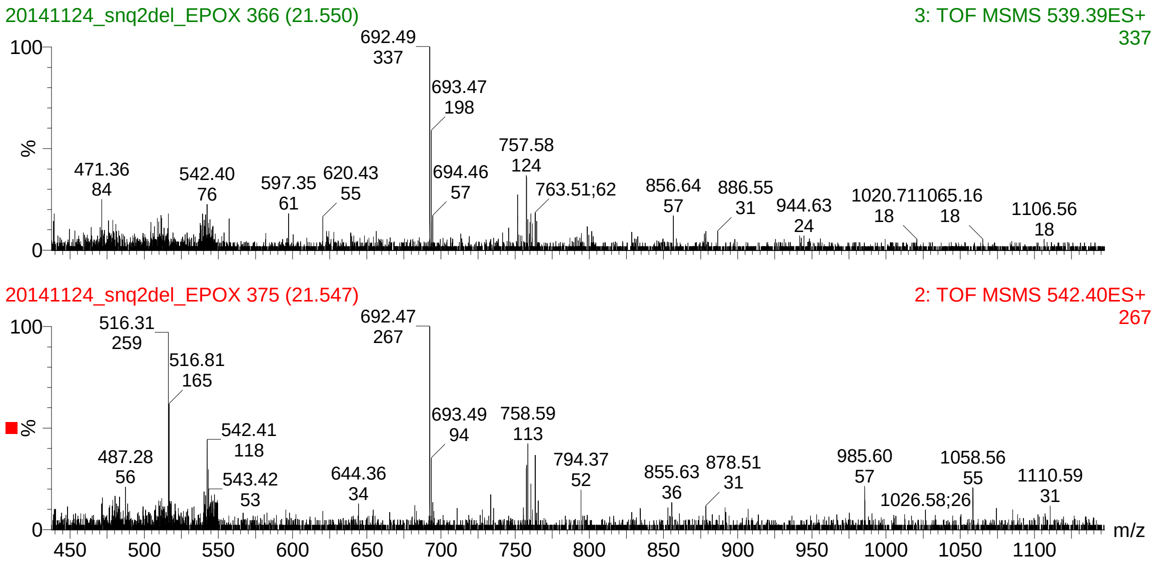

The Mascot report of the predicted fragment ions, error, and top hits is shown below.

Monoisotopic mass of neutral peptide Mr(calc): 1076.6957
Variable modifications:
K6 : GIST-Quat (K), with neutral loss 59.0735
Ions Score: 16 **Expect:** 1.3
Matches : 6/54 fragment ions using 22 most intense peaks (help)

#	b	b++	b*	b*++	Seq.	y	y++	y*	y*++	#
1	72.0444	36.5258			A					10
2	143.0815	72.0444			A	947.5924	474.2999	930.5659	465.7866	9
3	**256.1656**	128.5864			L	***876.5553***	438.7813	859.5288	430.2680	8
4	**327.2027**	164.1050			A	***763.4713***	382.2393	746.4447	373.7260	7
5	424.2554	212.6314			P	***692.4341***	346.7207	675.4076	338.2074	6
6	620.3766	310.6920	603.3501	302.1787	K	595.3814	298.1943	578.3548	289.6811	5
7	733.4607	367.2340	716.4341	358.7207	I	399.2602	200.1337			4
8	790.4822	395.7447	773.4556	387.2314	G	***286.1761***	143.5917			3
9	887.5349	444.2711	870.5084	435.7578	P	229.1547	115.0810			2
10					L	132.1019	66.5546			1

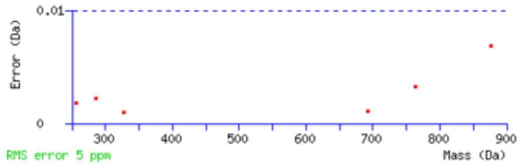

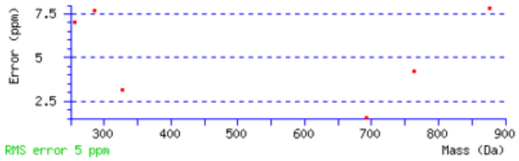

NCBI **BLAST** search of AALAPKIGPL
(Parameters: blastp, nr protein database, expect=20000, no filter, PAM30)
Other BLAST web gateways

All matches to this query

Score	Mr(calc)	Delta	Sequence
15.9	1076.6957	0.0008	AALAPKIGPL

Summary:

Although the Mascot scores are not very high, manual interpretation strongly supports the identification as AALAPKIGPL. All of the criteria have been met, and these criteria exclude the vast majority of false positives.

Similar analysis was done for every peptide listed in Table S1 for yeast peptides, as well as all peptides from human cell lines listed in Table S3.
