# Supplementary material for: Analysis of the Yeast Peptidome and Comparison with the Human Peptidome
Source: PLoS One. 2016 Sep 29;11(9):e0163312. doi: 10.1371/journal.pone.0163312 (PMC5042401; doi:10.1371/journal.pone.0163312)
Supplement: S2 Appendix — This Appendix shows an example of a peptide that has a “weak” Mascot score and which was rejected because it failed to meet the criteria. (PDF) [file pone.0163312.s002.pdf]

Appendix 2. Manual interpretation of Mascot results: Example of a peptide that does not match all of the criteria

Dasgupta et al, 2016

Overview: Every peptide tentatively identified by Mascot is manually reviewed and must match a number of criteria. While the Mascot score is a factor and those with scores above 50 are very likely to be correct, there is no rigid cut-off in score because some high-scoring peptides are clearly false positives (based on the criteria described in Appendix 1) while some peptides with moderate scores show convincing MS/MS spectra and match the criteria—these would be false negatives if the score was the only factor considered. Appendix 2 shows an example of a peptide that has a “weak” Mascot score, and which was **rejected** based on the criteria.

The Mascot Summary page (below and also in Appendix 1) shows the 38th through the 42nd ranked “hits” from a search of one sample (named “snqdel2_Epox”) against the yeast open reading frame database (6717 sequences; 3020751 residues), allowing for the D0-TMAB tags (named GIST in Mascot) on either the N-terminus or the Lys side chain and also allowing for the following variable modifications: Acetyl (N-term), Oxidation (M), and Cyano (C). Additional searches (not shown) were done with all other TMAB tags that are available on Mascot (i.e. the D3, D6, and D9 tags). Other key search parameters were: Peptide Mass Tolerance ± 0.01 Da; Fragment Mass Tolerance ± 0.2 Da; and Instrument type ESI-QUAD-TOF. The vast majority of the proteins with better Mascot scores had at least one peptide that passed the above criteria. For the peptides shown in the Mascot Summary below, only the peptide AALAPKIGPL from the protein 60S ribosomal protein L12A (i.e. the 38th ranked result) matched the criteria, as described in Appendix 1—the others failed to meet one or more criteria. In this Appendix, we show the details for one of these “rejected” peptides, LKDGPGMDIL from the SEN1 gene product (ranked 40th in the Mascot result indicated below).

38. YEL054C **Mass:** 17812 **Score:** 23 **Matches:** 2(0) **Sequences:** 1(0)
RPL12A SGDID:S000000780, Chr V from 53218-52721, reverse complement, Verified ORF, ""Protein component of the large (60S) ribosomal subunit, nearly identical to Rpl12Bp; rpl.

Query	Observed	Mr(expt)	Mr(calc)	Delta	Miss	Score	Expect	Rank	Unique	Peptide
190	539.3555	1076.6965	1076.6957	0.0008	0	(16)	1.3	1	U	S.AALAPKIGPL.G
311	602.9051	1203.7957	1203.7955	0.0003	0	22	0.23	1	U	S.AALAPKIGPL.G

Proteins matching the same set of peptides:
YDR418W **Mass:** 17812 **Score:** 23 **Matches:** 2(0) **Sequences:** 1(0)
RPL12B SGDID:S000002826, Chr IV from 1301609-1302106, Verified ORF, ""Protein component of the large (60S) ribosomal subunit, nearly identical to Rpl12Ap; rpl12a rpl12b doub.

39. YJL088W **Mass:** 37821 **Score:** 21 **Matches:** 2(0) **Sequences:** 2(0)
ARG3 SGDID:S000003624, Chr X from 268793-269809, Verified ORF, ""Ornithine carbamoyltransferase (carbamoylphosphate:L-ornithine carbamoyltransferase), catalyzes the sixth st

Query	Observed	Mr(expt)	Mr(calc)	Delta	Miss	Score	Expect	Rank	Unique	Peptide
531	700.3650	1398.7155	1398.7103	0.0052	0	21	5.8	1	U	M.STTASTPSSLRHL.I
671	765.4157	1528.8169	1528.8097	0.0072	0	2	5.9e+002	5	U	F.NISLDEVNKGINS.K

40. YLR430W **Mass:** 252339 **Score:** 19 **Matches:** 10(0) **Sequences:** 10(0)
SEN1 SGDID:S000004422, Chr XII from 993431-1000126, Verified ORF, ""Presumed helicase required for RNA polymerase II transcription termination and processing of RNAs; homolo

Query	Observed	Mr(expt)	Mr(calc)	Delta	Miss	Score	Expect	Rank	Unique	Peptide
38	446.7700	891.5255	891.5290	-0.0035	0	1	1.5e+002	9	U	A.KRFSNKL.T
100	492.3177	982.6208	982.6175	0.0033	0	9	43	1	U	P.LIQDIIIR.S
137	510.2790	1018.5435	1018.5481	-0.0045	0	13	46	6	U	L.ESCVRLIIS.T
435	656.8834	1311.7522	1311.7472	0.0050	0	19	7.7	1	U	R.LKDGPGMDIL.N
1264	574.3307	2293.2939	2293.3014	-0.0075	0	1	4.6e+002	3	U	S.LKSIPKACLMNSATALLRAV.L
1336	487.2825	2431.3763	2431.3758	0.0005	0	9	80	2	U	D.KKKNNKKAESPSTSSGTKKKS.S
1435	671.3445	2681.3488	2681.3404	0.0084	0	1	7.2e+002	10	U	A.HILAVSDIICSTLSGSAHDVLATMGI.K
1497	728.1312	2908.4955	2908.5005	-0.0049	0	0	1.1e+003	4	U	K.KSSIFGGMSVPSAVVPKTFPDVDSNKKA.A
1569	681.9425	3404.6761	3404.6666	0.0096	0	9	1.2e+002	1	U	T.LRSEIYCVKVMQMTTIEREYSTLEGLE.Y
1609	822.0036	4104.9816	4104.9779	0.0038	0	10	96	4	U	L.SKILADEDASQGFWSCIFSSDKHLYQAATNILYNT.F

41. YOR070C **Score:** 18 **Matches:** 1(0) **Sequences:** 1(0)
GYP1 SGDID:S000005596, Chr XV from 457822-455909, reverse complement, Verified ORF, "Cis-golgi GTPase-activating protein (GAP) for the Rab family members Ypt1p (in vivo) and

Query	Observed	Mr(expt)	Mr(calc)	Delta	Miss	Score	Expect	Rank	Unique	Peptide
38	446.7700	891.5255	891.5252	0.0004	0	18	2.9	1	U	V.CAAFLIK.W

42. YLR297W **Score:** 18 **Matches:** 1(0) **Sequences:** 1(0)
YLR297W SGDID:S000004288, Chr XII from 724046-724435, Uncharacterized ORF, "Putative protein of unknown function; green fluorescent protein (GFP)-fusion protein localizes to

Query	Observed	Mr(expt)	Mr(calc)	Delta	Miss	Score	Expect	Rank	Unique	Peptide
38	446.7700	891.5255	891.5178	0.0077	0	18	3	2	U	M.IFISLRSG.S

According to the above Mascot results, the peptide LKDGPGMDIL from the SEN1 gene product was tentatively identified from the 656.9 m/z ion, representing the peptide with 2 D0 isotopic tags—one on the N-terminus and the other on the internal Lys (see Mascot peptide view, below). The Mascot score of 19 was low, but other peptides with similar scores were subsequently found to match all of the criteria (see Appendix 1).

Peptide View

MS/MS Fragmentation of LKDGPGMDIL
Found in YLR430W in Yeast_ORF, SEN1 SGDID:S000004422, Chr XII from 993431-1000126, Verified ORF, ""Presumed helicase required for RNA polymerase II transcription termination and processing of RNAs; homolog of Senataxin which causes Ataxia-Oculomotor Apraxia 2 and a dominant form o

Match to Query 435: 1311.752248 from(656.883400,2+) intensity(161410.0000) scans(5259) rawscans(fn3ix303) rtinseconds(1206.099) index(1133)
Title: 1134: Scan 5259 (rt=1206.1, f=3, i=303) [C:\Users\Administrador\Desktop\Synapt_24_11_2014\20141124_snq2del_EPOX.raw]
Data file 20141124_snq2del_EPOX.mgf

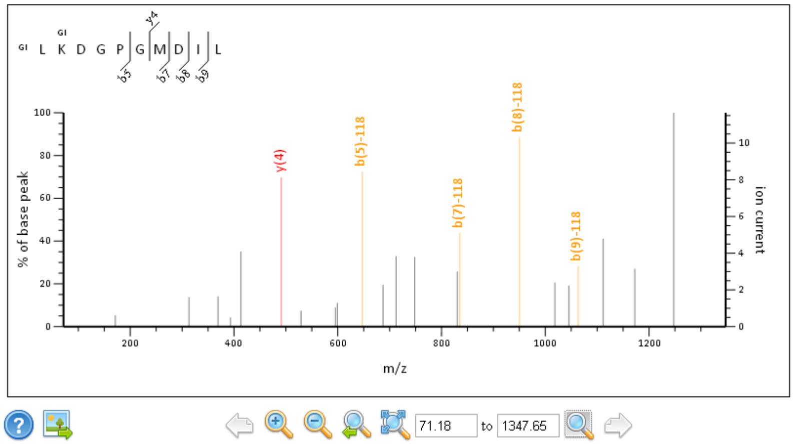

Label all possible matches ○ Label matches used for scoring ◉

Monoisotopic mass of neutral peptide Mr(calc): 1311.7472
Variable modifications:
N-term : GIST-Quat (N-term), with neutral loss 59.0735
K2 : GIST-Quat (K), with neutral loss 59.0735
Ions Score: 19 **Expect:** 7.7
Matches : 5/82 fragment ions using 11 most intense peaks (help)

The first step in manual evaluation of the data is to look at the MS spectra to see if the ion with m/z = 656.9 that eluted at 20.1’ is really a 2+ ion with 2 D0-TMAB tags.

The MS spectrum of the m/z 656.9 peak group (shown below) reveals that this ion is 2+. However, the 656.9 peak does **not** represent the peptide labeled with D0 (see MS spectra below). **Instead, the 656.9 ion is the D6 peak, and cannot possibly be the peptide assigned by Mascot. Furthermore, the mass difference between peaks is 3 Da, indicating 1 tag incorporated. Thus, criteria #1 and 2 are not met, and this is clearly a false positive.**

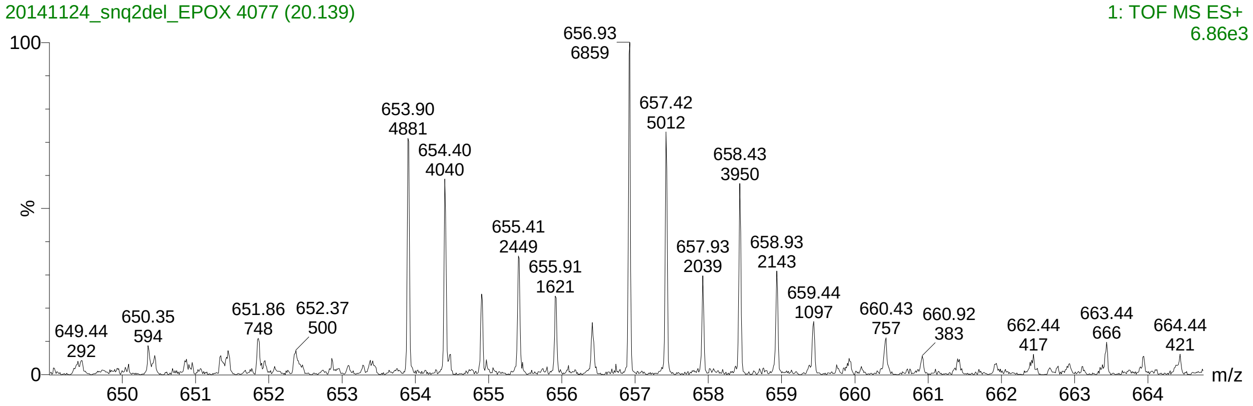

The failure to meet either criterion #1 or #2 is certainly enough to reject the Mascot hit, and in the normal evaluation process we would stop at this point. For this example we've included further evaluation to illustrate that false positives usually fail to meet most of the criteria.

Although the peptide proposed by Mascot is the top score (see below) which meets criteria 3, most of the other criteria are not met. The error for the fragments is higher than the "real" peptide (see Appendix 1, which was within 5 ppm). The b ion of 647.3 is assigned to cleavage of a Pro-Gly bond, but these are usually very weak (cleavage of Xaa-Pro bonds is strong, but Pro-Xaa cleavage is very weak). Although this latter point is not a rigid criterion, it raises a red flag about the identification.

#	b	b++	b*	b*++	b0	b0++	Seq.	y	y++	y*	y*++	y0	y0++	#
1	182.1176	91.5624					L							10
2	378.2387	189.6230	361.2122	181.1097			K	1013.4972	507.2522	996.4707	498.7390	995.4866	498.2470	9
3	493.2657	247.1365	476.2391	238.6232	475.2551	238.1312	D	817.3760	409.1917			799.3655	400.1864	8
4	550.2871	275.6472	533.2606	267.1339	532.2766	266.6419	G	702.3491	351.6782			684.3385	342.6729	7
5	***647.3399***	324.1736	630.3134	315.6603	629.3293	315.1683	P	645.3276	323.1675			627.3171	314.1622	6
6	704.3614	352.6843	687.3348	344.1710	686.3508	343.6790	G	548.2749	274.6411			530.2643	265.6358	5
7	***835.4019***	418.2046	818.3753	409.6913	817.3913	409.1993	M	**491.2534**	246.1303			473.2428	237.1251	4
8	***950.4288***	475.7180	933.4022	467.2048	932.4182	466.7128	D	360.2129	180.6101			342.2023	171.6048	3
9	***1063.5129***	532.2601	1046.4863	523.7468	1045.5023	523.2548	I	245.1860	123.0966					2
10							L	132.1019	66.5546					1

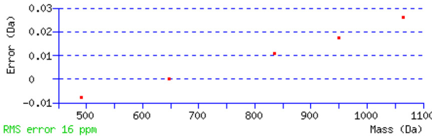

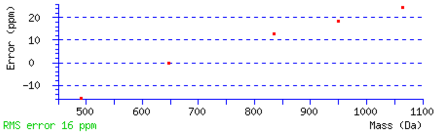

NCBI **BLAST** search of LKDGPGMDIL
(Parameters: blastp, nr protein database, expect=20000, no filter, PAM30)
Other BLAST web gateways

All matches to this query

Score	Mr(calc)	Delta	Sequence
19.0	1311.7472	0.0050	LKDGPGMDIL
2.8	1311.7479	0.0044	FFEFKDII
2.5	1311.7510	0.0012	DDNKRNALI
2.5	1311.7551	-0.0028	KGNEFHTIL
2.1	1311.7584	-0.0062	KVPKKVIDQCL
1.6	1311.7584	-0.0062	KLLGRMPED
1.6	1311.7510	0.0012	KGKKVQNGIDG
1.6	1311.7510	0.0012	KGKKVQNGIDG

Analysis of the MS/MS sequence for both the 653.9 and 656.9 ions (shown below) reveals that the majority of strong ions do not match the predicted fragment ions (listed above), and so criterion 4 is not met. Instead, the spectra are an excellent match to the peptide IAIDSTGVFKE from the protein Glyceraldehyde-3-phosphate dehydrogenase, isozyme 3, labeled with TMAB on the Lys but not on the N-terminus. This was identified by Mascot from the 653.9 ion in the search allowing D0-TMAB (Mascot Score 68) and from the 656.9 ion in the search allowing D6-TMAB (Mascot Score 35). Thus, it is absolutely clear that the Mascot “identification” of the 656.9 ion as the peptide LKDGPGMDIL is **not** correct, and this was clear from the manual inspection and the failure to meet the criteria.

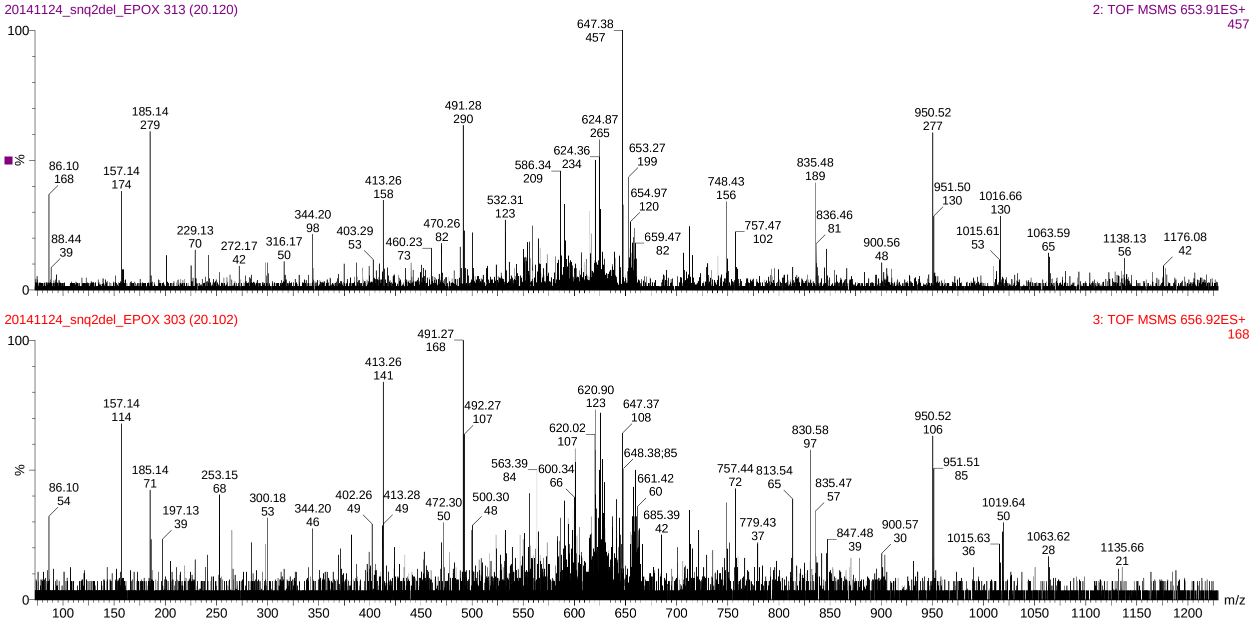

Summary:

Manual interpretation provides confidence that some peptides tentatively identified by Mascot are correct (as in Appendix 1), while others (as in this example) are clearly false positive. Others fall in between – they meet the most important criteria (matching the number and form of isotopic tags, charge, etc) but fail to meet all the criteria, such as five or more b or y ions (criterion #6). These are potentially correct but cannot be included with confidence based on the limited amount of MS/MS data, and so these are left off of the list of “identified” peptides. The bottom line—those peptides on our list meet the rigid criteria for identification listed in Appendix 1.
