# Supplementary material for: Analysis of the Yeast Peptidome and Comparison with the Human Peptidome
Source: PLoS One. 2016 Sep 29;11(9):e0163312. doi: 10.1371/journal.pone.0163312 (PMC5042401; doi:10.1371/journal.pone.0163312)

Figure S1. Schematic diagram of TMAB labeling strategy for each LC/MS run on yeast peptides. Four of the experiments started with two independent cultures each of wild-type (WT) or *pdr5*Δ or *snq2*Δ mutant yeast strains (top four panels). After growth to early log phase, each culture was split into two equal volumes; one of which was treated for one hour with the indicated proteasome inhibitor (bortezomib or epoxomicin, dissolved in DMSO), the other treated with a comparable amount of DMSO (maximum 0.1%). Thus, each experiment included two independent biological replicates for each control and treated strain. These four cultures were then processed for peptidomics as described in Materials and Methods. The labeling strategy for comparison of WT and *blm10*Δ strain yeast strains is shown in the lower panel. In all experiments, peptides were labeled with TMAB-NHS isotopic labels as indicated.

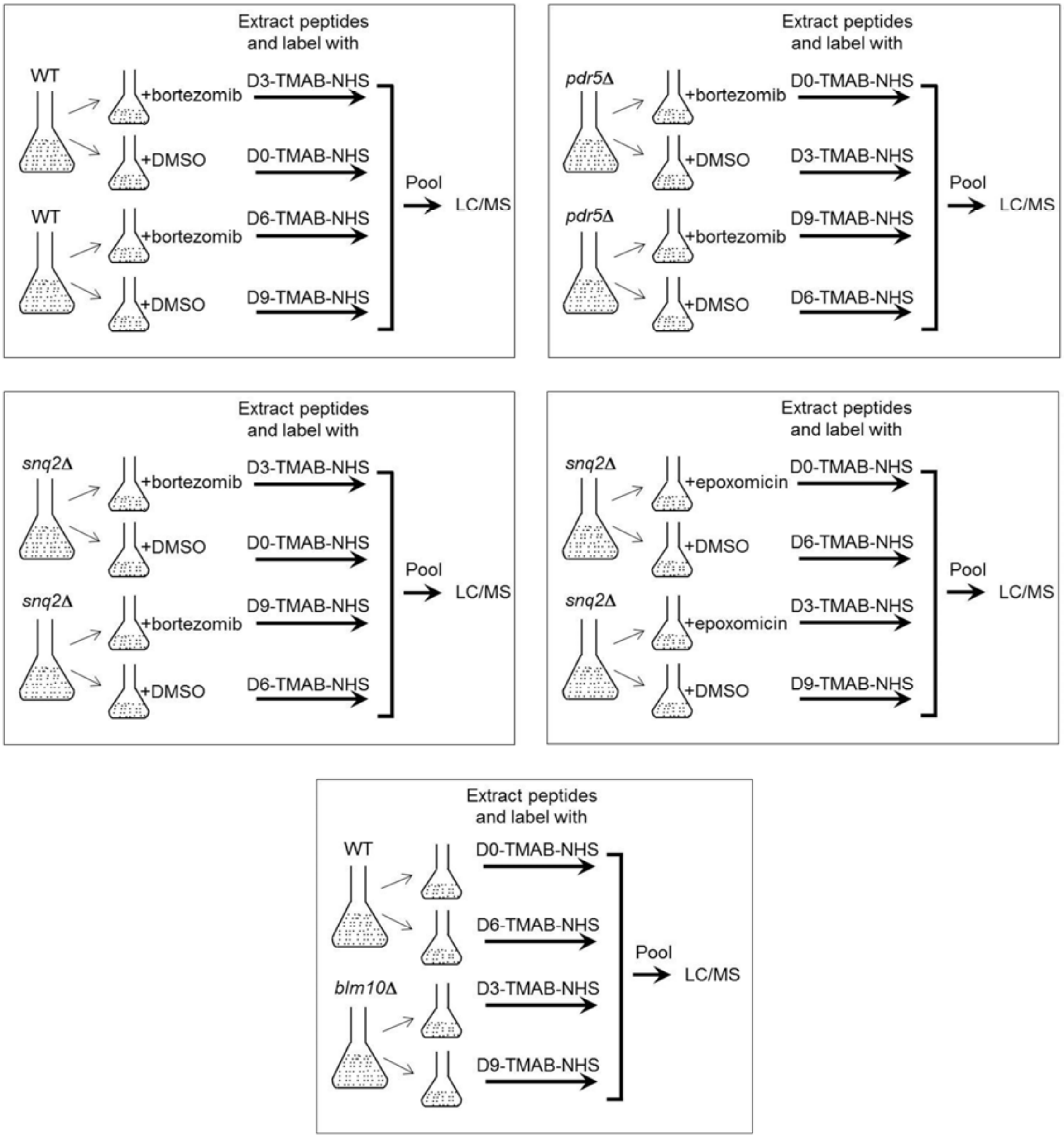

Supplement: S1 Fig — Four of the experiments started with two independent cultures each of wild-type (WT) or pdr5Δ or snq2Δ mutant yeast strains (top four panels). After growth to early log phase, each culture was split into two equal volumes; one of which was treated for one hour with the indicated proteasome inhibitor (bortezomib or epoxomicin, dissolved in DMSO), the other treated with a comparable amount of DMSO (maximum 0.1%). Thus, each experiment included two independent biological replicates for each control and treated strain. These four cultures were then processed for peptidomics as described in Materials and Methods. The labeling strategy for comparison of WT and blm10Δ strain yeast strains is shown in the lower panel. In all experiments, peptides were labeled with TMAB-NHS isotopic labels as indicated. (PDF) [file pone.0163312.s003.pdf]
