# Supplementary material for: Analysis of the Yeast Peptidome and Comparison with the Human Peptidome
Source: PLoS One. 2016 Sep 29;11(9):e0163312. doi: 10.1371/journal.pone.0163312 (PMC5042401; doi:10.1371/journal.pone.0163312)
Supplement: S2 Fig — The regions of the proteins that correspond to peptides found in the peptidome are indicated by underline. In some cases, multiple peptides were found and are indicated by a double underline. If more than 2 peptides found for a particular region of the protein, the additional peptides are not indicated. (PDF) [file pone.0163312.s004.pdf]

Figure S2. Alignments of proteins that are conserved between human and yeast. The regions of the proteins that correspond to peptides found in the peptidome are indicated by underline. In some cases, multiple peptides were found and are indicated by a double underline. If more than 2 peptides found for a particular region of the protein, the additional peptides are not indicated.

40S ribosomal protein S21 (RPS21A)

```
>gi|52783792|sp|P63220.1|RS21_HUMAN RecName: Full=40S ribosomal
protein S21
>sp|Q9CQR2|RS21_MOUSE 40S ribosomal protein S21 OS=Mus musculus
GN=Rps21
>sp|P0C0V8|RS21A_YEAST 40S ribosomal protein S21-A
OS=Saccharomyces cerevisiae (strain ATCC 204508 / S288c)
GN=RPS21A

Human MQNDAGEFVDLYVPRKCSASNRIIGAKDHASIQMNVAEVDKVTGRFNGQFKTYA
Mouse MQNDAGEFVDLYVPRKCSASNRIIAAKDHASIQMNVAEVDRTTGRFNGQFKTYG
Yeast MENDKGQLVELYVPRKCSATNRIIKADDHASVQINVAKVDEEGRAIPGEYVTYA

Human ICGAIRRMGESDDSILRLAKADGIVSKNF
Mouse ICGAIRRMGESDDSILRLAKADGIVSKNF
Yeast LSGYVRSRGESDDSLNRLAQNDGLLKNVWSYSR
```

60S acidic ribosomal protein P1 (RPP1A)

```
>sp|P05386|RLA1_HUMAN 60S acidic ribosomal protein P1 OS=Homo
sapiens GN=RPLP1
>tr|Q58E35|Q58E35_MOUSE 60S acidic ribosomal protein P1 OS=Mus
musculus GN=Rplp1
>sp|P05318|RLA1_YEAST 60S acidic ribosomal protein P1-alpha
OS=Saccharomyces cerevisiae (strain ATCC 204508/S288c) GN=RPP1A

Human  MASVSELACIYSALILHDDEVTVTEDKINALIKAAGVNVEPFWPGLFAKAL
Mouse  MASVSELACIYSALILHDDEVTVTEDKINALIKAAGVSVEPFWPGLFAKAL
Yeast  MSTESALS--YAALILADSEIEISSEKLLTLTNAANVPVENIWADIFAKAL

Human  ANVNIGSLICNVGAGGPAPAAGAAP-AGGPAPSTAAAPAEEKKVEAKKEES
Mouse  ANVNIGSLICNVGAGGPAPAAGAAP-AGGAAPSTAAAPAEEKKVEAKKEES
Yeast  DGQNLKDLLVNFSAGAAAPAGVAGGVAGGEAGEAEAEKEEE---EAK----

Human  EESDDDMGFGLFD
Mouse  EESEDDMGFGLFD
Yeast  EESDDDMGFGLFD
```

60S acidic ribosomal protein P2 (RPP2A and RPP2B)

>gi|133061|sp|P05387.1|RLA2_HUMAN RecName: Full=60S acidic ribosomal protein P2.
>sp|P99027|RLA2_MOUSE 60S acidic ribosomal protein P2 OS=Mus musculus GN=Rplp2
>sp|P05319|RLA2_YEAST 60S acidic ribosomal protein P2-alpha OS=Saccharomyces cerevisiae (strain ATCC 204508 / S288c) GN=RPP2A
>sp|P02400|RLA4_YEAST 60S acidic ribosomal protein P2-beta OS=Saccharomyces cerevisiae (strain ATCC 204508/S288c) GN=RPP2B

```
Human       MRYVASYLLAALGGNSSPSAKDIKKILDSVGIEADDDRLNKVISELN
Mouse       MRYVASYLLAALGGNSSPSAKDIKKILDSVGIEADDDRLNKVISELN
Yeast-alpha MKYLAAYLLLNAAGNT-PDATKIKAILESVGIEIEDEKVSSVLSALE
Yeast-beta  MKYLAAYLLLVQGGNAAPSAADIKAVVESVGAEVDEARINELLSSLE

Human       GKNIEDVIAQGIGKLASVPAGGAVAVSAAPGSAAPAAGSAPAAAEEK
Mouse       GKNIEDVIAQGVGKLASVPAGGAVAVSAAPGSAAPAAGSAPAAAEEK
Yeast-alpha GKSVDELITEGNEKLAAVPAAGPASAGGAAAASGDAAA-----EEEK
Yeast-beta  GKGSLEEIIAEGQKKFATVPTGGASSAAAGAAGAAAGGDAA--EEEK

Human       KDEKKEESEESDDDMGFGLFD
Mouse       KDEKKEESEESDDDMGFGLFD
Yeast-alpha –EE--EAAEESDDDMGFGLFD
Yeast-beta  -EE--EAKEESDDDMGFGLFD
```

60S ribosomal protein L31 (RPL31A)

>gi|51702807|sp|P62899.1|RL31_HUMAN RecName: Full=60S ribosomal protein L31
>sp|P62900|RL31_MOUSE 60S ribosomal protein L31 OS=Mus musculus GN=Rpl31
>sp|P0C2H8|RL31A_YEAST 60S ribosomal protein L31-A OS=Saccharomyces cerevisiae (strain ATCC 204508/S288c) GN=RPL31A

```
Human  MAPAKKGGEKKKGRSAINEVVTREYTINIHKRIHGVGFKKRAPRALKEIRKFAMK
Mouse  MAPAKKGGEKKKGRSAINEVVTREYTINIHKRIHGVGFKKRAPRALKEIRKFAMK
Yeast              MAGLKDVVTREYTINLHKRLHGVSFKKRAPRAVKEIKKFAKL

Human  EMGTPDVRIDTRLNKAVWAKGIRNVPYRIRVRLSRKRNEDEDSPNKLYTLVTYVP
Mouse  EMGTPDVRIDTRLNKAVWAKGIRNVPYRIRVRLSRKRNEDEDSPNKLYTLVTYVP
Yeast  HMGTDDVRLAPELNQAIWKRGVKGVEYRLRLRISRKRNEEEDAKNPLFSYVEPVL

Human  VTTFKNLQTVNVDEN
Mouse  VTTFKNLQTVNVDEN
Yeast  VASAKGLQTVVVEEDA
```

Acyl-CoA-binding protein/ Diazepam-binding inhibitor (ACB1)

>gi|118276|sp|P07108.2|ACBP_HUMAN RecName: Full=Acyl-CoA-binding protein; Short=ACBP; AltName: Diazepam-binding inhibitor; DBI.
>sp|P31786|ACBP_MOUSE Acyl-CoA-binding protein OS=Mus musculus GN=Dbi
>sp|P31787|ACBP_YEAST Acyl-CoA-binding protein OS=Saccharomyces cerevisiae (strain ATCC 204508/S288c) GN=ACB1

```
Human  MSQAEFEKAAEEVRHLKTKPSDEEMLFIYGHYKQATVGDINTERPGMLDFT
Mouse  MSQAEFDKAAEEVKRLKTQPTDEEMLFIYSHFKQATVGDVNTDRPGLLDLK
Yeast  MVSQLFEEKAKAVNELPTKPSTDELLELYALYKQATVGDNDKEKPGIFNMK

Human  GKAKWDAWNELKGTSKEDAMKAYINKVEELKKKYGI
Mouse  GKAKWDSWNKLKGTSKESAMKTYVEKVDELKKKYGI
Yeast  DRYKWEAWENLKGKSQEDAEKEYIALVDQLIAKYSS
```

Calmodulin (CMD1)

>gi|49037474|sp|P62158.2|CALM_HUMAN RecName: Full=Calmodulin;
>sp|P62204|CALM_MOUSE Calmodulin OS=Mus musculus GN=Calm1
>sp|P06787|CALM_YEAST Calmodulin OS=Saccharomyces cerevisiae (strain ATCC 204508/S288c) GN=CMD1

```
Human  MADQLTEEQIAEFKEAFSLFDKDGDGTITTKELGTVMRSLGQNPTEAELQDM
Mouse  MADQLTEEQIAEFKEAFSLFDKDGDGTITTKELGTVMRSLGQNPTEAELQDM
Yeast  MSSNLTEEQIAEFKEAFALFDKDNNGSISSSELATVMRSLGLSPSEAEVNDL

Human  INEVDADGNGTIDFPEFLTMMARKMKDTDSEEEIREAFRVFDKDGNGYISAA
Mouse  INEVDADGNGTIDFPEFLTMMARKMKDTDSEEEIREAFRVFDKDGNGYISAA
Yeast  MNEIDVDGNHQIEFSEFLALMSRQLKSNDSEQELLEAFKVFDKNGDGLISAA

Human  ELRHVMTNLGEKLTDEEVDEMIREADIDGDGQVNYEEFVQMMTAK
Mouse  ELRHVMTNLGEKLTDEEVDEMIREADIDGDGQVNYEEFVQMMTAK
Yeast  ELKHVLTSIGEKLTDAEVDDMLREV--SDGSGEINIQQFAALLSK
```

Cathepsin D (PEP4)

>gi|115717|sp|P07339.1|CATD_HUMAN RecName: Full=Cathepsin D;
>sp|P18242|CATD_MOUSE Cathepsin D OS=Mus musculus GN=Ctsd
>tr|A6ZW99|A6ZW99_YEAS7 Vacuolar proteinase A OS=Saccharomyces cerevisiae (strain YJM789) GN=PEP4

```
Human MQP-----SSLLPLALCLLAAPASALVRIPLHKFTSIRRTMSEVGGSVED-------
Mouse MKT-----PGVLLLILGLLASSSFAIIRIPLRKFTSIRRTMTEVGGSVED-------
Yeast MFSLKALLPLALLLVSANQVAAKVHKAKIYKHELSDEMKEVTFEQHLAHLGQKYLTQ
```

```
Human LIAKGPVSKYSQAVPAVTEGPIPEVLKNYMDAQYYGEIGIGTPPQCFTVVFDTGSSN
Mouse LILKGPITKYSMQSSPKTTEPVSELLKNYLDAQYYGDIGIGTPPQCFTVVFDTGSSN
Yeast FEKANPEVVFSREHPFFTEGGHDVPLTNYLNAQYYTDITLGTPPQNFKVILDTGSSN

Human LWVPSIHCKLLDIACWIHHKYNSDKSSTYVKNGTSFDIHYGSGSLSGYLSQDTVSVP
Mouse LWVPSIHCKILDIACWVHHKYNSDKSSTYVKNGTSFDIHYGSGSLSGYLSQDTVSVP
Yeast LWVPSNECGS--LACFLHSKYDHEASSSYKANGTEFAIQYGTGSLEGYISQDTLSI-

Human CQSASSASALGGVKVERQVFGEATKQPGITFIAAKFDGILGMAYPRISVNNVLPVFD
Mouse CKSDQ--SKARGIKVEKQIFGEATKQPGIVFVAAKFDGILGMGYPHISVNNVLPVFD
Yeast ----------GDLTIPKQDFAEATSEPGLTFAFGKFDGILGLGYDTISVDKVVPPFY

Human NLMQQKLVDQNIFSFYLS-RDPDAQPGGELMLGGTDSKYYKGSLSYLNVTRKAYWQV
Mouse NLMQQKLVDKNIFSFYLN-RDPEGQPGGELMLGGTDSKYYHGELSYLNVTRKAYWQV
Yeast NAIQQDLLDEKRFAFYLGDTSKDTENGGEATFGGIDESKFKGDITWLPVRRKAYWEV

Human HLDQVEVASGLTLCKEGCEAIVDTGTSLMVGPVDEVRELQKAIGAVPLIQGEYMIPC
Mouse HMDQLEVGNELTLCKGGCEAIVDTGTSLLVGPVEEVKELQKAIGAVPLIQGEYMIPC
Yeast KFEGIGLGDEYAELESHGAAI-DTGTSLITLPSGLAEMINAEIGAKKGWTGQYTLDC

Human EKVSTLPAITLKLGGKGYKLSPEDYTLKVSQAGKTLCLSGFMGMDIPPPSGPLWILG
Mouse EKVSSLPTVYLKLGGKNYELHPDKYILKVSQGGKTICLSGFMGMDIPPPSGPLWILG
Yeast NTRDNLPDLIFNFNGYNFTIGPYDYTLEVSGSCISAIT----PMDFPEPVGPLAIVG

Human DVFIGRYYTVFDRDNNRVGFAEAARL
Mouse DVFIGSYYTVFDRDNNRVGFANAVVL
Yeast DAFLRKYYSIYDLGNNAVGLAKAI
```

Enolase 1 and 2 (ENO1, ENO2)

>sp|P06733|ENOA_HUMAN Alpha-enolase OS=Homo sapiens GN=ENO1
>gi|49168648|emb|CAG38819.1| ENO2 [Homo sapiens]
>sp|P17182|ENOA_MOUSE Alpha-enolase OS=Mus musculus GN=Eno1
>gi|2289903|gb|AAC36002.1| ENO2 [Mus musculus]
>sp|P00924|ENO1_YEAST Enolase 1 OS=Saccharomyces cerevisiae GN=ENO1
>gi|285810079|tpg|DAA06866.1| TPA: phosphopyruvate hydratase ENO2

```
Human1 MSILKIHAREIFDSRGNPTVEVDLFTSKGLFRAAVPSGASTGIYEALELRDNDKTRYMGKGVS
Human2 MSIEKIWAREILDSRGNPTVEVDLYTAKGLFRAAVPSGASTGIYEALELRDGDKQRYLGKGVL
Mouse1 MSILRIHAREIFDSRGNPTVEVDLYTAKGLFRAAVPSGASTGIYEALELRDNDKTRFMGKGVS
Mouse2 MSIEKIWAREILDSRGNPTVEVDLYTAKGLFRAAVPSGASTGIYEALELRDGDKQRYLGKGVL
Yeast1 MAVSKVYARSVYDSRGNPTVEVELTTEKGVFRSIVPSGASTGVHEALEMRDGDKSKWMGKGVL
Yeast2 MAVSKVYARSVYDSRGNPTVEVELTTEKGVFRSIVPSGASTGVHEALEMRDEDKSKWMGKGVM

Human1 KAVEHINKTIAPALVSKKLNVTEQEKIDKLMIEMDGTENKSKFGANAILGVSLAVCKAGAVEK
Human2 KAVDHINSTIAPALISSGLSVVEQEKLDNLMLELDGTENKSKFGANAILGVSLAVCKAGAAER
Mouse1 QAVEHINKTIAPALVSKKVNVVEQEKIDKLMIEMDGTENKSKFGANAILGVSLAVCKAGAVEK
Mouse2 KAVDHINSRIAPALISSGISVVEQEKLDNLMLELDGTENKSKFGANAILGVSLAVCKAGAAER
Yeast1 HAVKNVNDVIAPAFVKANIDVKDQKAVDDFLISLDGTANKSKLGANAILGVSLAASRAAAAEK
Yeast2 NAVNNVNNVIAAAFVKANLDVKDQKAVDDFLLSLDGTANKSKLGANAILGVSMAAARAAAAEK
```

Human1 GVPLYRHIADLAGNSEVILPVPA-F-NVINGGSHAGNKLAMQEFMILPVGAANFREAMRIGAE
Human2 ELPLYRHIAQLAGNSDLILPVPA-F-NVINGGSHAGNKLAMQEFMILPVGAESFRDAMRLGAE
Mouse1 GVPLYRHIADLAGNPEVILPVPA-F-NVINGGSHAGNKLAMQEFMILPVGASSFREAMRIGAE
Mouse2 DLPLYRHIAQLAGNSDLILPVPA-F-NVINGGSHAGNKLAMQEFMILPVGAESFRDAMRLGAE
Yeast1 NVPLYKHLADLSKSKTSPYVLPVPFLNVLNGGSHAGGALALQEFMIAPTGAKTFAEALRIGSE
Yeast2 NVPLYQHLADLSKSKTSPYVLPVPFLNVLNGGSHAGGALALQEFMIAPTGAKTFAEAMRIGSE

Human1 VYHNLKNVIKEKYGKDATNVGDEGGFAPNILENKEGLELLKTAIGKAGYTDKVVIGMDVAASE
Human2 VYHTLKGVIKDKYGKDATNVGDEGGFAPNILENSEALELVKEAIDKAGYTEKIVIGMDVAASE
Mouse1 VYHNLKNVIKEKYGKDATNVGDEGGFAPNILENKEALELLKTAIAKAGYTDQVVIGMDVAASE
Mouse2 VYHTLKGVIKDKYGKDATNVGDEGGFAPNILENSEALELVKEAIDKAGYTEKMVIGMDVAASE
Yeast1 VYHNLKSLTKKRYGASAGNVGDEGGVAPNIQTAEEALDLIVDAIKAAGHDGKIKIGLDCASSE
Yeast2 VYHNLKSLTKKRYGASAGNVGDEGGVAPNIQTAEEALDLIVDAIKAAGHDGKVKIGLDCASSE

Human1 FFRSGKYDLDFKSPD-DPSRYISPDQLADLYKSFIKDYPVVSIEDPFDQDDWGAWQKFTASAG
Human2 FYRDGKYDLDFKSPT-DPSRYITGDQLGALYQDFVRDYPVVSIEDPFDQDDWAAWSKFTANVG
Mouse1 FYRSGKYDLDFKSPD-DPSRYITPDQLADLYKSFVQNYPVVSIEDPFDQDDWGAWQKFTASAG
Mouse2 FYRDGKYDLDFKSPA-DPSRYITGDQLGALYQDFVRNYPVVSIEDPFDQDDWAAWSKFTANVG
Yeast1 FFKDGKYDLDFKNPNSDKSKWLTGPQLADLYHSLMKRYPIVSIEDPFAEDDWEAWSHFFKTAG
Yeast2 FFKDGKYDLDFKNPESDKSKWLTGVELADMYHSLMKRYPIVSIEDPFAEDDWEAWSHFFKTAG

Human1 IQVVGDDLTVTNPKRIAKAVNEKSCNCLLLKVNQIGSVTESLQACKLAQANGWGVMVSHRSGE
Human2 IQIVGDDLTVTNPKRIERAVEEKACNCLLLKVNQIGSVTEAIQACKLAQENGWGVMVSHRSGE
Mouse1 IQVVGDDLTVTNPKRIAKAASEKSCNCLLLKVNQIGSVTESLQACKLAQSNGWGVMVSHRSGE
Mouse2 IQIVGDDLTVTNPKRIERAVEEKACNCLLLKVNQIGSVTEAIQACKLAQENGWGVMVSHRSGE
Yeast1 IQIVADDLTVTNPKRIATAIEKKAADALLLKVNQIGTLSESIKAAQDSFAAGWGVMVSHRSGE
Yeast2 IQIVADDLTVTNPARIATAIEKKAADALLLKVNQIGTLSESIKAAQDSFAANWGVMVSHRSGE

Human1 TEDTFIADLVVGLCTGQIKTGAPCRSERLAKYNQLLRIEEELGSKAKFAGRNFRNPLAK
Human2 TEDTFIADLVVGLCTGQIKTGAPCRSERLAKYNQLMRIEEELGDEARFAGHNFRNPSVL
Mouse1 TEDTFIADLVVGLCTGQIKTGAPCRSERLAKYNQILRIEEELGSKAKFAGRSFRNPLAK
Mouse2 TEDTFIADLVVGLCTGQIKTGAPCRSERLAKYNQLMRIEEELGDEARFAGHNFRNPSVL
Yeast1 TEDTFIADLVVGLRTGQIKTGAPARSERLAKLNQLLRIEEELGDNAVFAGENFHHGDKL
Yeast2 TEDTFIADLVVGLRTGQIKTGAPARSERLAKLNQLLRIEEELGDKAVYAGENFHHGDKL

Heat shock 70 kDa protein 1A/Heat shock cognate 71 kDa protein (SSA2)

>sp|P11142|HSP7C_HUMAN Heat shock cognate 71 kDa protein OS=Homo sapiens GN=HSPA8
>sp|P63017|HSP7C_MOUSE Heat shock cognate 71 kDa protein OS=Mus musculus GN=Hspa8
>sp|P10592|HSP72_YEAST Heat shock protein SSA2 OS=Saccharomyces cerevisiae (strain ATCC 204508 / S288c) GN=SSA2

Human   MSKGPAVGIDLGTTYSCVGVFQHGKVEIIANDQGNRTTPSYVAFTDTERLIGD
Mouse   MSKGPAVGIDLGTTYSCVGVFQHGKVEIIANDQGNRTTPSYVAFTDTERLIGD
Yeast   MSK--AVGIDLGTTYSCVAHFSNDRVDIIANDQGNRTTPSFVGFTDTERLIGD

```
human AAKNQVAMNPTNTVFDAKRLIGRRFDDAVVQSDMKHWPFMVVNDAGRPKVQVEYKGE
mouse AAKNQVAMNPTNTVFDAKRLIGRRFDDAVVQSDMKHWPFMVVNDAGRPKVQVEYKGE
yeast AAKNQAAMNPANTVFDAKRLIGRNFNDPEVQGDMKHFPFKLIDVDGKPQIQVEFKGE

human TKSFYPEEVSSMVLTKMKEIAEAYLGKTVTNAVVTVPAYFNDSQRQATKDAGTIAGL
mouse TKSFYPEEVSSMVLTKMKEIAEAYLGKTVTNAVVTVPAYFNDSQRQATKDAGTIAGL
yeast TKNFTPEQISSMVLGKMKETAESYLGAKVNDAVVTVPAYFNDSQRQATKDAGTIAGL

human NVLRIINEPTAAAIAYGLDKKVGAERNVLIFDLGGGTFDVSILTIEDGIFEVKSTAG
mouse NVLRIINEPTAAAIAYGLDKKVGAERNVLIFDLGGGTFDVSILTIEDGIFEVKSTAG
yeast NVLRIINEPTAAAIAYGLDKK-GKEEHVLIFDLGGGTFDVSLLSIEDGIFEVKATAG

human DTHLGGEDFDNRMVNHFIAEFKRKHKKDISENKRAVRRLRTACERAKRTLSSSTQAS
mouse DTHLGGEDFDNRMVNHFIAEFKRKHKKDISENKRAVRRLRTACERAKRTLSSSTQAS
yeast DTHLGGEDFDNRLVNHFIQEFKRKNKKDLSTNQRALRRLRTACERAKRTLSSSAQTS

human IEIDSLYEGIDFYTSITRARFEELNADLFRGTLDPVEKALRDAKLDKSQIHDIVLVG
mouse IEIDSLYEGIDFYTSITRARFEELNADLFRGTLDPVEKALRDAKLDKSQIHDIVLVG
yeast VEIDSLFEGIDFYTSITRARFEELCADLFRSTLDPVEKVLRDAKLDKSQVDEIVLVG

human GSTRIPKIQKLLQDFFNGKELNKSINPDEAVAYGAAVQAAILSGDKSENVQDLLLLD
mouse GSTRIPKIQKLLQDFFNGKELNKSINPDEAVAYGAAVQAAILSGDKSENVQDLLLLD
yeast GSTRIPKVQKLVTDYFNGKEPNRSINPDEAVAYGAAVQAAILTGDESSKTQDLLLLD

human VTPLSLGIETAGGVMTVLIKRNTTIPTKQTQTFTTYSDNQPGVLIQVYEGERAMTKD
mouse VTPLSLGIETAGGVMTVLIKRNTTIPTKQTQTFTTYSDNQPGVLIQVYEGERAMTKD
yeast VAPLSLGIETAGGVMTKLIPRNSTIPTKKSEVFSTYADNQPGVLIQVFEGERAKTKD

human NNLLGKFELTGIPPAPRGVPQIEVTFDIDANGILNVSAVDKSTGKENKITITNDKGR
mouse NNLLGKFELTGIPPAPRGVPQIEVTFDIDANGILNVSAVDKSTGKENKITITNDKGR
yeast NNLLGKFELSGIPPAPRGVPQIEVTFDVDSNGILNVSAVEKGTGKSNKITITNDKGR

human LSKEDIERMVQEAEKYKAEDEKQRDKVSSKNSLESYAFNMKATVEDEKLQGKINDED
mouse LSKEDIERMVQEAEKYKAEDEKQRDKVSSKNSLESYAFNMKATVEDEKLQGKINDED
yeast LSKEDIEKMVAEAEKFKEEDEKESQRIASKNQLESIAYSLKNTISEAGDKLEQADKD

human KQKILDKCNEIINWLDKNQTAEKEEFEHQQKELEKVCNPIITKLYQSAGGM-PGGMP
mouse KQKILDKCNEIISWLDKNQTAEKEEFEHQQKELEKVCNPIITKLYQSAGGM-PGGMP
yeast AVTKKAE--ETIAWLDSNTTATKEEFDDQLKELQEVANPIMSKLYQAGGAPE-GAAP

human GGFPGGGAPPSGGASSGPTIEEVD
mouse GGFPGGGAPPSGGASSGPTIEEVD
yeast GGFPGG-APPAPEAE-GPTVEEVD
```

Heat shock protein HSP 90-alpha (HSC82, HSP82)

>gi|92090606|sp|P07900.5|HS90A_HUMAN RecName: Full=Heat shock protein HSP 90-alpha; AltName: Full=Heat shock 86 kDa;
>sp|P07901|HS90A_MOUSE Heat shock protein HSP 90-alpha OS=Mus musculus GN=Hsp90aa1
>sp|P15108|HSC82_YEAST ATP-dependent molecular chaperone HSC82 OS=Saccharomyces cerevisiae (strain ATCC 204508/S288c) GN=HSC82
>sp|P02829|HSP82_YEAST ATP-dependent molecular chaperone HSP82 OS=Saccharomyces cerevisiae (strain ATCC 204508/S288c) GN=HSP82

```
Human    MPEETQTQDQPMEEEEVETFAFQAEIAQLMSLIINTFYSNKEIFLRELISNSSDAL
Mouse    MPEETQTQDQPMEEEEVETFAFQAEIAQLMSLIINTFYSNKEIFLRELISNSSDAL
Yeast HSC82           MAGETFEFQAEITQLMSLIINTVYSNKEIFLRELISNASDAL
Yeast HSP82           MASETFEFQAEITQLMSLIINTVYSNKEIFLRELISNASDAL

Human  DKIRYESLTDPSKLDSGKELHINLIPNKQDRTLTIVDTGIGMTKADLINNLGTIAKS
Mouse  DKIRYESLTDPSKLDSGKELHINLIPSKQDRTLTIVDTGIGMTKADLINNLGTIAKS
YeastC DKIRYQALSDPKQLETEPDLFIRITPKPEEKVLEIRDSGIGMTKAELINNLGTIAKS
YeastP DKIRYKSLSDPKQLETEPDLFIRITPKPEQKVLEIRDSGIGMTKAELINNLGTIAKS

Human  GTKAFMEALQAGADISMIGQFGVGFYSAYLVAEKVTVITKHNDDEQYAWESSAGGSF
Mouse  GTKAFMEALQAGADISMIGQFGVGFYSAYLVAEKVTVITKHNDDEQYAWESSAGGSF
YeastC GTKAFMEALSAGADVSMIGQFGVGFYSLFLVADRVQVISKNNEDEQYIWESNAGGSF
YeastP GTKAFMEALSAGADVSMIGQFGVGFYSLFLVADRVQVISKSNDDEQYIWESNAGGSF

Human  TVRTDTG-EPMGRGTKVILHLKEDQTEYLEERRIKEIVKKHSQFIGYPITLFVEKER
Mouse  TVRTDTG-EPMGRGTKVILHLKEDQTEYLEERRIKEIVKKHSQFIGYPITLFVEKER
YeastC TVTLDEVNERIGRGTVLRLFLKDDQLEYLEEKRIKEVIKRHSEFVAYPIQLLVTKEV
YeastP TVTLDEVNERIGRGTILRLFLKDDQLEYLEEKRIKEVIKRHSEFVAYPIQLVVTKEV

Human DKEVSDDEAEEKEDKEEEKEKEEKESEDKPEIEDVGSDEEEE-KKDGDKKKKKKIKEK
Mouse DKEVSDDEAEEKEEKEEEKEKEEKESDDKPEIEDVGSDEEEEEKKDGDKKKKKKIKEK
Yeast EKEVPIPEEEKKDEEKKDEDD------KKPKLEEV--DEEEEEKKPKTKKVKEE----
Yeast EKEVPIPEEEKKDEEKKDEEKKDEDD-KKPKLEEV--DEEEEKK-PKTKKVKEE----

Human  YIDQEELNKTKPIWTRNPDDITNEEYGEFYKSLTNDWEDHLAVKHFSVEGQLEFRAL
Mouse  YIDQEELNKTKPIWTRNPDDITNEEYGEFYKSLTNDWEEHLAVKHFSVEGQLEFRAL
YeastC VQELEELNKTKPLWTRNPSDITQEEYNAFYKSISNDWEDPLYVKHFSVEGQLEFRAI
YeastP VQEIEELNKTKPLWTRNPSDITQEEYNAFYKSISNDWEDPLYVKHFSVEGQLEFRAI

Human  LFVPRRAPFDLFENRKKKNNIKLYVRRVFIMDNCEELIPEYLNFIRGVVDSEDLPLN
Mouse  LFVPRRAPFDLFENRKKKNNIKLYVRRVFIMDNCEELIPEYLNFIRGVVDSEDLPLN
YeastC LFIPKRAPFDLFESKKKKNNIKLYVRRVFITDEAEDLIPEWLSFVKGVVDSEDLPLN
YeastP LFIPKRAPFDLFESKKKKNNIKLYVRRVFITDEAEDLIPEWLSFVKGVVDSEDLPLN
```

```
Human  ISREMLQQSKILKVIRKNLVKKCLELFTELAEDKENYKKFYEQFSKNIKLGIHEDSQ
Mouse  ISREMLQQSKILKVIRKNLVKKCLELFTELAEDKENYKKFYEQFSKNIKLGIHEDSQ
YeastC LSREMLQQNKIMKVIRKNIVKKLIEAFNEIAEDSEQFDKFYSAFAKNIKLGVHEDTQ
YeastP LSREMLQQNKIMKVIRKNIVKKLIEAFNEIAEDSEQFEKFYSAFSKNIKLGVHEDTQ

Human  NRKKLSELLRYYTSASGDEMVSLKDYCTRMKENQKHIYYITGETKDQVANSAFVERL
Mouse  NRKKLSELLRYYTSASGDEMVSLKDYCTRMKENQKHIYFITGETKDQVANSAFVERL
YeastC NRAALAKLLRYNSTKSVDELTSLTDYVTRMPEHQKNIYYITGESLKAVEKSPFLDAL
YeastP NRAALAKLLRYNSTKSVDELTSLTDYVTRMPEHQKNIYYITGESLKAVEKSPFLDAL

Human  RKHGLEVIYMIEPIDEYCVQQLKEFEGKTLVSVTKEGLELPEDEEEKKKQEEKKTKF
Mouse  RKHGLEVIYMIEPIDEYCVQQLKEFEGKTLVSVTKEGLELPEDEEEKKKQEEKKTKF
YeastC KAKNFEVLFLTDPIDEYAFTQLKEFEGKTLVDITKDFELEETDEEKAEREKEIKEYE
YeastP KAKNFEVLFLTDPIDEYAFTQLKEFEGKTLVDITKDFELEETDEEKAEREKEIKEYE

Human  ENLCKIMKDILEKKVEKVVVSNRLVTSPCCIVTSTYGWTANMERIMKAQALRDNSTM
Mouse  ENLCKIMKDILEKKVEKVVVSNRLVTSPCCIVTSTYGWTANMERIMKAQALRDNSTM
YeastC PLTKALKDILGDQVEKVVVSYKLLDA-PAAIRTGQFGWSANMERIMKAQALRD-SSM
YeastP PLTKALKEILGDQVEKVVVSYKLLDA-PAAIRTGQFGWSANMERIMKAQALRD-SSM

Human  MGYMAAKKHLEINPDHSIIETLRQKAEADK-NDKSVKDLVILLYETALLSSGFSLED
Mouse  MGYMAAKKHLEINPDHSIIETLRQKAEADK-NDKSVKDLVILLYETALLSSGFSLED
YeastC SSYMSSKKTFEISPKSPIIKELKKRVDEGGAQDKTVKDLTNLLFETALLTSGFSLEE
YeastP SSYMSSKKTFEISPKSPIIKELKKRVDEGGAQDKTVKDLTKLLYETALLTSGFSLDE

Human  PQTHANRIYRMIKLGLGIDEDDPTADDTSAAVTEEMPPLEGDDDTSRMEEVD
Mouse  PQTHANRIYRMIKLGLGIDEDDPTVDDTSAAVTEEMPPLEGDDDTSRMEEVD
YeastC PTSFASRINRLISLGLNIDEDEETETAPEASTEAPVEEVPA—DT--EMEEVD
YeastP PTSFASRINRLISLGLNIDEDEETETAPEASTAAPVEEVPA-DT--EMEEVD
```

---

10 kDa heat shock protein (HSP10)

>gi|4504523|ref|NP_002148.1| 10 kDa heat shock protein, mitochondrial [Homo sapiens]
>gi|6680309|ref|NP_032329.1| 10 kDa heat shock protein, mitochondrial [Mus musculus]
Yeast HSP10   YOR020C   10 kDa heat shock protein, mitochondrial(HSP10) (10 kDa chaperonin)

```
Human MAGQAFR-KFL-PLFDRVLVERSAAETVTKGGIMLPEKSQGKVLQATVVAVGSG
Mouse MAGQAFR-KFL-PLFDRVLVERSAAETVTKGGIMLPEKSQGKVLQATVVAVGSG
Yeast MSTLLKSAKSIVPLMDRVLVQRIKAQAKTASGLYLPEKNVEKLNQAEVVAVGPG

Human SKGKGGEIQPVSVKVGDKVLLPEYGG-TKVVLDDKDYFLFRDGDILGKYV-D
Mouse GKGKSGEIEPVSVKVGDKVLLPEYGG-TKVVLDDKDYFLFRDSDILGKYV-D
Yeast FTDANGNKVVPQVKVGDQVLIPQFGGSTIKLGNDDEVILFRDAEILAKIAKD
```

Hsp90 co-chaperone / co-chaperone protein (SBA1)

>gi|8928247|sp|Q15185.1|TEBP_HUMAN RecName: Full=Prostaglandin E synthase 3; AltName: Full=Cytosolic prostaglandin E2 synthase; Short=cPGES; AltName: Full=Hsp90 co-chaperone
>sp|Q9R0Q7|TEBP_MOUSE Prostaglandin E synthase 3 OS=Mus musculus GN=Ptges3
>sp|P28707|SBA1_YEAST Co-chaperone protein SBA1 OS=Saccharomyces cerevisiae (strain ATCC 204508 / S288c) GN=SBA1

```
Human            MQPASAKWYDRRDYVFIEFCVEDSKDVNVNFEKSKLTFSCLGGS
Mouse            MQPASAKWYDRRDYVFIEFCVEDSKDVNVNFEKSKLTFSCLGGS
Yeast MSDKVINPQVAWAQRSSTTDPERNYVLITVSIADCDAPELTIKPSYIELKAQSKP

Human ---D-NFKHLN-EIDLFHCIDPNDSKHKRTDRSILCC-LRKGE-SGQSWPRLTKE
Mouse ---D-NFKHLN-EIDLFHCIDPNDSKHKRTDRSILCC-LRKGE-SGQSWPRLTKE
Yeast HVGDENVHHYQLHIDLYKEIIPEKTMHKVANGQHYFLKLYKKDLESEYWPRLTKE

Human RAKLNWLSVDFNNWKDWEDDSDEDMS--NFDR---FSEMMNNMGGDEDVDLPEVD
Mouse RAKLNWLSVDFNNWKDWEDDSDEDMS--NFDR---FSEMMDHMGGDEDVDLPEVD
Yeast KVKYPYIKTDFDKWVDEDEQDEVEAEGNDAAQGMDFSQMMGGAGGAGGAGGMDFS

Human GADDDSQDSDDEKMPDLE
Mouse GADDDSQDSDDEKMPDLE
Yeast QMMGGAGGAGSPDMAQLQQLLAQSGGNLDMGDFKENDEEDEEEEIEPEVKA
```

Peroxiredoxin-1 (TSA1)

>gi|548453|sp|Q06830.1|PRDX1_HUMAN RecName: Full=Peroxiredoxin-1; AltName: Full=Natural killer cell-enhancing factor A; Short=NKEF-A
>sp|P35700|PRDX1_MOUSE Peroxiredoxin-1 OS=Mus musculus GN=Prdx1
>sp|P34760|TSA1_YEAST Peroxiredoxin TSA1 OS=Saccharomyces cerevisiae (strain ATCC 204508 / S288c) GN=TSA1

```
Human MSSGNAKIGHPAPNFKATAVMPDGQFKDISLSDYKGKYVVFFFYPLDFTFVCPTEII
Mouse MSSGNAKIGYPAPNFKATAVMPDGQFKDISLSEYKGKYVVFFFYPLDFTFVCPTEII
Yeast MVAQVQK---QAPTFKKTAVV-DGVFDEVSLDKYKGKYVVLAFIPLAFTFVCPTEII

Human AFSDRAEEFKKLNCQVIGASVDSHFCHLAWVNTPKKQGGLGPMNIPLVSDPKRTIAQ
Mouse AFSDRADEFKKLNCQVIGASVDSHFCHLAWINTPKKQGGLGPMNIPLISDPKRTIAQ
Yeast AFSEAAKKFEEQGAQVLFASTDSEYSLLAWTNIPRKEGGLGPINIPLLADTNHSLSR

Human DYGVLKADEGISFRGLFIIDDKGILRQITVNDLPVGRSVDETLRLVQAFQFTDKHGE
Mouse DYGVLKADEGISFRGLFIIDDKGILRQITINDLPVGRSVDEIIRLVQAFQFTDKHGE
Yeast DYGVLIEEEGVALRGLFIIDPKGVIRHITINDLPVGRNVDEALRLVEAFQWTDKNGT
```

Human VCPAGWKPGSDTIKPDVQKSKEYFSK-QK
Mouse VCPAGWKPGSDTIKPDVNKSKEYFSK-QK
Yeast VLPCNWTPGAATIKPTVEDSKEYFEAANK

Peroxiredoxin-5 (AHP1)

>gi|317373539|sp|P30044.4|PRDX5_HUMAN RecName: Full=Peroxiredoxin-5, mitochondrial; AltName: Full=Alu corepressor 1
>sp|P99029|PRDX5_MOUSE Peroxiredoxin-5, mitochondrial OS=Mus musculus GN=Prdx5
>gi|1709682|sp|P38013.4|AHP1_YEAST RecName: Full=Peroxiredoxin type-2; AltName: Full=AHPC1

Human MGLAGVCALRRSAGYILVGGAGGQSAAAAARRYSEGEWASGGVRSFSRAAAAMAPIK
Mouse   MLQLGLRVLGCKASSVLRASTCLAGRAGRK--EAGWECGGARSFSSSAVTMAPIK
Yeast   MSDLVNKKFPAGDYKFQYIAISQSDADSESC-K

Human VGDAIPAVEVFEGEPGNKVNLAELFKGKKGVLFGVPGAFTPGCSKTHLPGFVEQAEA
Mouse VGDAIPSVEVFEGEPGKKVNLAELFKGKKGVLFGVPGAFTPGCSKTHLPGFVEQAGA
Yeast -MPQTVEWSKLCKMPQTVEWSKLISENKKVIITGAPAAFSPTCTVSHIPGYINYLDE

Human L-KAKGV-QVVACLSVNDAFVTGEWGR--AHKAEGKVRLLADPTGAFGKETDLLLDDS
Mouse L-KAKGA-QVVACLSVNDVFVIEEWGR--AHQAEGKVRLLADPTGAFGKATDLLLDDS
Yeast LVKEKEVDQVIVVTVDNPFANQA-WAKSLGVKDTTHIKFASDPGCAFTKSIGF---E-

Human LVSIFGNRRLKRFSMVVQDGIVKALNVE-PDGTGLTCSLAPNIISQL
Mouse LVSLFGNRRLKRFSMVIDNGIVKALNVE-PDGTGLTCSLAPNILSQL
Yeast LAVGDGVYWSGRWAMVVENGIVTYAAKETNPGTDVTVSSVESVLAHL

Phosphoglycerate mutase (GPM1)

>gi|130353|sp|P15259.3|PGAM2_HUMAN RecName: Full= Phosphoglycerate mutase 2; AltName: Full=BPG-dependent PGAM 2; AltName: Full=Muscle-specific phosphoglycerate mutase; AltName:
>sp|O70250|PGAM2_MOUSE Phosphoglycerate mutase 2 OS=Mus musculus GN=Pgam2
>sp|P00950|PMG1_YEAST Phosphoglycerate mutase 1 OS=Saccharomyces cerevisiae (strain ATCC 204508 / S288c) GN=GPM1

Human 2 MATHRLVMVRHGESTWNQENRFCGWFDAELSEKGTEEAKRGAKAIKDAKMEFD
Mouse 2 MTTHRLVMVRHGESLWNQENRFCGWFDAELSEKGAEEAKRGATAIKDAKIEFD
Yeast   M--PKLVLVRHGQSEWNEKNLFTGWVDVKLSAKGQQEAARAGELLKEKKVYPD

Human 2 ICYTSVLKRAIRTLWAILDGTDQMWLPVVRTWRLNERHYGGLTGLNKAETAAK
Mouse 2 ICYTSVLKRAIRTLWTILDVTDQMWVPVVRTWRLNERHYGGLTGLNKAETAAK
Yeast   VLYTSKLSRAIQTANIALEKADRLWIPVNRSWRLNERHYGDLQGKDKAETLKK

Human 2 HGEEQVKIWRRSFDIPPPPMDEKHPYYNSISKERRYAGLKPGELPTCESLKDT
Mouse 2 HGEEQVKIWRRSFDTPPPPMDEKHNYYTSISKDRRYAGLKPEELPTCESLKDT
Yeast   FGEEKFNTYRRSFDVPPPPIDASSPFSQKGDERYKYVDPNV--LPETESLALV

Human 2 IARALPFWNEEIVPQIKAGKRVLIAAHGNSLRGIVKHLEGMSDQAIMELNLPT
Mouse 2 IARALPFWNEEIAPKIKAGQRVLIAAHGNSLRGIVKHLEGMSDQAIMELNLPT
Yeast   IDRLLPYWQDVIAKDLLSGKTVMIAAHGNSLRGLVKHLEGISDADIAKLNIPT

Human 2 GIPIVYELNKELKPTKPMQFLGDEETVRKAMEAVAAQGKAK
Mouse 2 GIPIVYELDQNLKPTKPMRFLGDEETVRKAMEAVAAQGKAK
Yeast   GIPLVFELDENLKPSKPSYYLDPEAAAAGAA-AVANQGK-K

Pyruvate Kinase M / Yeast pyruvate kinase 1 (CDC19)

>gi|20178296|sp|P14618.4|KPYM_HUMAN RecName: Full=Pyruvate kinase PKM
>sp|P52480|KPYM_MOUSE Pyruvate kinase PKM OS=Mus musculus GN=Pkm
>sp|P00549|KPYK1_YEAST Pyruvate kinase 1 OS=Saccharomyces cerevisiae (strain ATCC 204508/S288c) GN=CDC19

Human MSKPHSEAGTAFIQTQQLHAAMADTFLEHMCRLDIDSPPITARNTGIICTIGPAS
Mouse MPKPHSEAGTAFIQTQQLHAAMADTFLEHMCRLDIDSAPITARNTGIICTIGPAS
Yeast MSRLERLTSLNVVAGSDLRR-----------------------TSIIGTIGPKT

Human RSVETLKEMIKSGMNVARLNFSHGTHEYHAETIKNVRTATESFASDPILYRPVAV
Mouse RSVEMLKEMIKSGMNVARLNFSHGTHEYHAETIKNVREATESFASDPILYRPVAV
Yeast NNPETLVALRKAGLNIVRMNFSHGSYEYHKSVIDNARKSEELYPGRPL-----AI

Human ALDTKGPEIRTGLIKGSGTAEVELKKGATLKITLDNAYMEKCDENILWLDYKNIC
Mouse ALDTKGPEIRTGLIKGSGTAEVELKKGATLKITLDNAYMEKCDENILWLDYKNIC
Yeast ALDTKGPEIRTGTTTNDVDYPIPPNHEMIF—TTDD-KYAKACDDKIMYVDYKNIT

Human KVVEVGSKIYVDDGLISLQVKQKGADFLVTEVENGGS-LGSKKGVNLPGAAVDLPA
Mouse KVVEVGSKIYVDDGLISLQVKEKGADFLVTEVENGGS-LGSKKGVNLPGAAVDLPA
Yeast KVISAGRIIYVDDGVLSFQVLEVVDDKTLKVKALNAGKICSHKGVNLPGTDVDLPA

Human VSEKDIQDLKFGVEQDVDMVFASFIRKASDVHEVRKVLGEKGKNIKIISKIENHE
Mouse VSEKDIQDLKFGVEQDVDMVFASFIRKAADVHEVRKVLGEKGKNIKIISKIENHE
Yeast LSEKDKEDLRFGVKNGVHMVFASFIRTANDVLTIREVLGEQGKDVKIIVKIENQQ

Human GVRRFDEILEASDGIMVARGDLGIEIPAEKVFLAQKMMIGRCNRAGKPVICATQM
Mouse GVRRFDEILEASDGIMVARGDLGIEIPAEKVFLAQKMMIGRCNRAGKPVICATQM
Yeast GVNNFDEILKVTDGVMVARGDLGIEIPAPEVLAVQKKLIAKSNLAGKPVICATQM

Human LESMIKKPRPTRAEGSDVANAVLDGADCIMLSGETAKGDYPLEAVRMQHLIAREA
Mouse LESMIKKPRPTRAEGSDVANAVLDGADCIMLSGETAKGDYPLEAVRMQHLIAREA
Yeast LESMTYNPRPTRAEVSDVGNAILDGADCVMLSGETAKGNYPINAVTTMAETAVIA

Human EAAIYHLQLFEELRRLAPITSDPTEATAVGAVEASFKCCSGAIIVLTKSGRSAHQ
Mouse EAAIYHLQLFEELRRLAPITSDPTEAAAVGAVEASFKCCSGAIIVLTKSGRSAHQ
Yeast EQAIAYLPNYDDMRNCTPKPTSTTETVAASAVAAVFEQKAKAIIVLSTSGTTPRL

Human VARYRPRAPIIAVTRNPQTARQAHLYRGIFPVLCKDPVQEAWAEDVDLRVNFAMN
Mouse VARYRPRAPIIAVTRNPQTARQAHLYRGIFPVLCKDAVLNAWAEDVDLRVNLAMD
Yeast VSKYRPNCPIILVTRCPRAARFSHLYRGVFPFVFEKEPVSDWTDDVEARINFGIE

Human VGKARGFFKKGDVVIVLTGWRPGSGFTNTMRVVPVP
Mouse VGKARGFFKKGDVVIVLTGWRPGSGFTNTMRVVPVP
Yeast KAKEFGILKKGDTYVSIQGFKAGAGHSNTLQVSTV

Stress-70 protein, mitochondrial (SSC1)

>sp|P38646|GRP75_HUMAN Stress-70 protein, mitochondrial OS=Homo sapiens GN=HSPA9
>sp|P38647|GRP75_MOUSE Stress-70 protein, mitochondrial OS=Mus musculus GN=Hspa9
>sp|P0CS90|HSP77_YEAST Heat shock protein SSC1, mitochondrial OS=Saccharomyces cerevisiae (strain ATCC 204508/S288c) GN=SSC1

Note: italics indicates region of protein known to be cleaved

Human *MISASRAAAARLVGAAASRGPTAARHQDSWNGLSHEAFRLVSRRDY*ASEAIKGAVVG
Mouse *MISASRAAAARLVGTAASRSPAAARPQDGWNGLSHEAFRFVSRRDY*ASEAIKGAVVG
Yeast *MLAAKNILNRSSLSSSFRIATRL*Q-----------------------STKVQGSVIG

Human IDLGTTNSCVAVMEGKQAKVLENAEGARTTPSVVAFTADGERLVGMPAKRQAVTNPN
Mouse IDLGTTNSCVAVMEGKQAKVLENAEGARTTPSVVAFTADGERLVGMPAKRQAVTNPN
Yeast IDLGTTNSAVAIMEGKVPKIIENAEGSRTTPSVVAFTKEGERLVGIPAKRQAVVNPE

Human NTFYATKRLIGRRYDDPEVQKDIKNVPFKIVRASNGDAWVEAHGKLYSPSQIGAFVL
Mouse NTFYATKRLIGRRYDDPEVQKDTKNVPFKIVRASNGDAWVEAHGKLYSPSQIGAFVL
Yeast NTLFATKRLIGRRFEDAEVQRDIKQVPYKIVKHSNGDAWVEARGQTYSPAQIGGFVL

Human MKMKETAENYLGHTAKNAVITVPAYFNDSQRQATKDAGQISGLNVLRVINEPTAAAL
Mouse MKMKETAENYLGHTAKNAVITVPAYFNDSQRQATKDAGQISGLNVLRVINEPTAAAL
Yeast NKMKETAEAYLGKPVKNAVVTVPAYFNDSQRQATKDAGQIVGLNVLRVVNEPTAAAL

Human AYGLDKSEDKVIAVYDLGGGTFDISILEIQKGVFEVKSTNGDTFLGGEDFDQALLRH
Mouse AYGLDKSEDKVIAVYDLGGGTFDISILEIQKGVFEVKSTNGDTFLGGEDFDQALLRH
Yeast AYGLEKSDSKVVAVFDLGGGTFDISILDIDNGVFEVKSTNGDTHLGGEDFDIYLLRE

```
Human IVKEFKRETGVDLTKDNMALQRVREAAEKAKCELSSSVQTDINLPYLTMDSSGPKHL
Mouse IVKEFKRETGVDLTKDNMALQRVREAAEKAKCELSSSVQTDINLPYLTMDASGPKHL
Yeast IVSRFKTETGIDLENDRMAIQRIREAAEKAKIELSSTVSTEINLPFITADASGPKHI

Human NMKLTRAQFEGIVTDLIRRTIAPCQKAMQDAEVSKSDIGEVILVGGMTRMPKVQQTV
Mouse NMKLTRAQFEGIVTDLIKRTIAPCQKAMQDAEVSKSDIGEVILVGGMTRMPKVQQTV
Yeast NMKFSRAQFETLTAPLVKRTVDPVKKALKDAGLSTSDISEVLLVGGMSRMPKVVETV

Human QDLFGRAPSKAVNPDEAVAIGAAIQGGVLAGDVTDVLLLDVTPLSLGIETLGGVFTK
Mouse QDLFGRAPSKAVNPDEAVAIGAAIQGGVLAGDVTDVLLLDVTPLSLGIETLGGVFTK
Yeast KSLFGKDPSKAVNPDEAVAIGAAVQGAVLSGEVTDVLLLDVTPLSLGIETLGGVFTR

Human LINRNTTIPTKKSQVFSTAADGQTQVEIKVCQGEREMAGDNKLLGQFTLIGIPPAPR
Mouse LINRNTTIPTKKSQVFSTAADGQTQVEIKVCQGEREMAGDNKLLGQFTLIGIPPAPR
Yeast LIPRNTTIPTKKSQIFSTAAAGQTSVEIRVFQGERELVRDNKLIGNFTLAGIPPAPK

Human GVPQIEVTFDIDANGIVHVSAKDKGTGREQQIVIQSSGGLSKDDIENMVKNAEKYAE
Mouse GVPQIEVTFDIDANGIVHVSAKDKGTGREQQIVIQSSGGLSKDDIENMVKNAEKYAE
Yeast GVPQIEVTFDIDADGIINVSARDKATNKDSSITVAGSSGLSENEIEQMVNDAEKFKS

Human EDRRKKERVEAVNMAEGIIHDTETKMEEFKDQLPADECNKLKEEISKMRELLARKDS
Mouse EDRRKKERVEAVNMAEGIIHDTETKMEEFKDQLPADECNKLKEEISKMRALLAGKDS
Yeast QDEARKQAIETANKADQLANDTENSLKEFEGKVDKAEAQKVRDQITSLKELVARVQG

Human ETGENIRQAASS---LQQASLKLFEMAYKKMASEREGSGSSGTGEQKEDQKEEKQ
Mouse ETGENIRQAASS---LQQASLKLFEMAYKKMASEREGSGSSGTGEQKEDQKEEKQ
Yeast GEEVNAEELKTKTEELQTSSMKLFEQLYKNDSNNNNNNNGNNA-----ESGETKQ
```

Stress-induced-phosphoprotein 1 / Heat shock protein (STI1)

```
>gi|400042|sp|P31948.1|STIP1_HUMAN RecName: Full=Stress-induced-
phosphoprotein 1; Short=STI1
>sp|Q60864|STIP1_MOUSE Stress-induced-phosphoprotein 1 OS=Mus
musculus GN=Stip1
>sp|P15705|STI1_YEAST Heat shock protein STI1 OS=Saccharomyces
cerevisiae (strain ATCC 204508 / S288c) GN=STI1

Human MEQVNELKEKGNKALSVGNIDDALQCYSE-AIKLDPH-NHVLYSNRSAAYAKKGDYQK
Mouse MEQVNELKEKGNKALSAGNIDDALQCYSE-AIKLDPQ-NHVLYSNRSAAYAKKGDYQK
Yeast MSLTADEYKQQGNAAFTAKDYDKAIELFTKAIEVSETPNHVLYSNRSACYTSLKKFSD

Human AYEDGCKTVDLKPDWGKGYSRKAAALEFLNRFEEAKRTYEEGLKHEANNPQLKEGL--
Mouse AYEDGCKTVDLKPDWGKGYSRKAAALEFLNRFEEAKRTYEEGLKHEANNLQLKEGL--
Yeast ALNDANECVKINPSWSKGYNRLGAAHLGLGDLDEAESNYKKALELDASNKAAKEGLDQ
```

Human --QNMEARLAER--KFMNPFNMPNLYQKLESDPRTRTLLSDPTYRELIEQLRNKPSDL
Mouse --QNMEARLAER--KFMNPFNLPNLYQKLENDPRTRSLLSDPTYRELIEQLQNKPSDL
Yeast VHRTQQARQAQPDLGLTQLFADPNLIENLKKNPKTSEMMKDPQLVAKLIGYKQNPQAI

Human GTKLQ--PRIMTTLSVLLGVDLG--------SM--------------------DEEEE
Mouse GTKLQ-DPRVMTTLSVLLGVDLG--------SM--------------------DEEEE
Yeast GQDLFTDPRLMTIMATLMGVDLNMDDINQSNSMPKEPETSKSTEQKKDAEPQSDSTTS

Human -ATPPPPPPPKKETKPEPMEEDLPENKKQALKEKELGNDAYKKKDFDTALKHYDKAKE
Mouse AATPPPPPPPKKEPKPEPMEEDLPENKKQALKEKELGNDAYKKKDFDKALKHYDRAKE
Yeast KENSSKAPQKEESKESEPMEVDEDDSKIEADKEKAEGNKFYKARQFDEAIEHYNKAWE

Human LDPTNMTYITNQAAVYFEKGDYNKCRELCEKAIEVGRENREDYRQIAKAYARIGNSYF
Mouse LDPTNMTYITNQAAVHFEKGDYNKCRELCEKAIEVGRENREDYRQIAKAYARIGNSYF
Yeast LHKDITYLNNRAAAEY-EKGEYETAISTLNDAVEQGREMRADYKVISKSFARIGNAYH

Human KEEKYKDAIHFYNKSLAEHRTPDVLKKCQQAEKILKEQERLAYINPDLALEEKNKGNE
Mouse KEEKYKDAIHFYNKSLAEHRTPDVLKKCQQAEKILKEQERLAYINPDLALEEKNKGNE
Yeast KLGDLKKTIEYYQKSLTEHRTADILTKLRNAEKELKKAEAEAYVNPEKAEEARLEGKE

Human CFQKGDYPQAMKHYTEAIKRNPKDAKLYSNRAACYTKLLEFQLALKDCEECIQLEPTF
Mouse CFQKGDYPQAMKHYTEAIKRNPRDAKLYSNRAACYTKLLEFQLALKDCEECIQLEPTF
Yeast YFTKSDWPNAVKAYTEMIKRAPEDARGYSNRAAALAKLMSFPEAIADCNKAIEKDPNF

Human IKGYTRKAAALEAMKDYTKAMDVYQKALDLD------SSCKEAADGYQRCMMAQY---
Mouse IKGYTRKAAALEAMKDYTKAMDVYQKALDLD------SSCKEAADGYQRCMMAQY---
Yeast VRAYIRKATAQIAVKEYASALETLDAARTKDAEVNNGSSAREIDQLYYKASQQRFQPG

Human NRHDSPEDVKRRAMADPEVQQIMSDPAMRLILEQMQKDPQALSEHLKNPVIAQKIQKL
Mouse NRHDSPEDVKRRAMADPEVQQIMSDPAMRLILEQMQKDPQALSEHLKNPVIAQKIQKL
Yeast TSNETPEETYQRAMKDPEVAAIMQDPVMQSILQQAQQNPAALQEHMKNPEVFKKIQTL

Human MDVGLIAI-R
Mouse MDVGLIAI-R
Yeast IAAGIIRTGR

Small ubiquitin-related modifier 2 (SUMO2) / Ubiquitin-like protein SMT3 (SMT3)

>gi|378405233|sp|P61956.3|SUMO2_HUMAN RecName: Full=Small ubiquitin-related modifier 2; Short=SUMO-2; AltName: Full=HSMT3
>sp|P61957|SUMO2_MOUSE Small ubiquitin-related modifier 2 OS=Mus musculus GN=Sumo2
>sp|Q12306|SMT3_YEAST Ubiquitin-like protein SMT3 OS=Saccharomyces cerevisiae (strain ATCC 204508 / S288c) GN=SMT3

Human MAD------EKPKEGVKTENNDHINLKVAGQDGSVVQFKIKRHTPLSKLMKAYCERQG
Mouse MA------DEKPKEGVKTENNDHINLKVAGQDGSVVQFKIKRHTPLSKLMKAYCERQG
Yeast MSDSEVNQEAKPEVKPEVKPETHINLKVSDGSSEIF-FKIKKTTPLRRLMEAFAKRQG

Hum2 LSMRQIRFRFDGQPINETDTPAQLEMEDEDTIDVFQQQTGGVY
Mou2 LSMRQIRFRFDGQPINETDTPAQLEMEDEDTIDVFQQQTGGVY
Yst KEMDSLRFLYDGIRIQADQTPEDLDMEDNDIIEAHREQIGGATY

Superoxide dismutase (SOD1)

>sp|P00441|SODC_HUMAN Superoxide dismutase [Cu-Zn] OS=Homo sapiens GN=SOD1
>sp|P08228|SODC_MOUSE Superoxide dismutase [Cu-Zn] OS=Mus musculus GN=Sod1
>sp|P00445|SODC_YEAST Superoxide dismutase [Cu-Zn] OS=Saccharomyces cerevisiae (strain ATCC 204508 / S288c) GN=SOD1

Human MATKAVCVLKGDGPVQGIINFEQKESNGPVKVWGSIKGLTEGL-HGFHVHEFG
Mouse MAMKAVCVLKGDGPVQGTIHFEQKASGEPVVLSGQITGLTEGQ-HGFHVHQYG
Yeast MVQ-AVAVLKGDAGVSGVVKFEQASESEPTTVSYEIAGNSPNAERGFHIHEFG

Human DNTAGCTSAGPHFNPLSRKHGGPKDEERHVGDLGNVTADKDGVADVSIEDSVI
Mouse DNTQGCTSAGPHFNPHSKKHGGPADEERHVGDLGNVTAGKDGVANVSIEDRVI
Yeast DATNGCVSAGPHFNPFKKTHGAPTDEVRHVGDMGNVKTDENGVAKGSFKDSLI

Human SLSGDHCIIGRTLVVHEKADDLGKGGNEESTKTGNAGSRLACGVIGIAQ
Mouse SLSGEHSIIGRTMVVHEKQDDLGKGGNEESTKTGNAGSRLACGVIGIAQ
Yeast KLIGPTSVVGRSVVIHAGQDDLGKGDTEESLKTGNAGPRPACGVIGLTN

Thioredoxin (TRX2)

>gi|135773|sp|P10599.3|THIO_HUMAN RecName: Full=Thioredoxin; Short=Trx
>sp|P10639|THIO_MOUSE Thioredoxin OS=Mus musculus GN=Txn
>sp|P22803|TRX2_YEAST Thioredoxin-2 OS=Saccharomyces cerevisiae (strain ATCC 204508 / S288c) GN=TRX2

Human MVKQIESKTAFQEALDAAGDKLVVVDFSATWCGPCKMIKPFFHSLSEKYSNVIF
Mouse MVKLIESKEAFQEALAAAGDKLVVVDFSATWCGPCKMIKPFFHSLCDKYSNVVF
Yeast MVTQLKSASEYDSALASGDKLVVVDFF-ATWCGPCKMIAPMIEKFAEQYSDAAF

Human LEVDVDDCQDVASECEVKCMPTFQFFKKGQKVGEFSGANKEKLEATINELV
Mouse LEVDVDDCQDVAADCEVKCMPTFQFYKKGQKVGEFSGANKEKLEASITEYA
Yeast YKLDVDEVSDVAQKAEVSSMPTLIFYKGGKEVTRVVGANPAAIKQAIASNV

Triosephosphate isomerase (TPI1)

>sp|P60174|TPIS_HUMAN Triosephosphate isomerase OS=Homo sapiens GN=TPI1
>sp|P17751|TPIS_MOUSE Triosephosphate isomerase OS=Mus musculus GN=Tpi1
>sp|P00942|TPIS_YEAST Triosephosphate isomerase OS=Saccharomyces cerevisiae (strain ATCC 204508 / S288c) GN=TPI1

```
h M---AE----------DGEEAEFHFAALYISGQWPRLRADTDLQRLGSSAMAPSRKFFVGGN
m MEGKAEQQGAGLTMAEGGEKEEFCFTAIYISGQWREPCVCTDLQRLEPGTMAPTRKFFVGGN
y                                                   MA--RTFFVGGN

h WKMNGRKQSLGELIGTLNAAKVPADTEVVCAPPTAYIDF-ARQKLDPKIAVAAQNCYKVTNG
m WKMNGRKKCLGELICTLNAANVPAGTEVVCAPPTAYIDF-ARQKLDPKIAVAAQNCYKVTNG
y FKLNGSKQSIKEIVERLNTASIPENVEVVICPPATYLDYSVSLVKKPQVTVGAQNAYLKASG

h AFTGEISPGMIKDCGATWVVLGHSERRHVFGESDELIGQKVAHALAEGLGVIACIGEKLDER
m AFTGEISPGMIKDLGATWVVLGHSERRHVFGESDELIGQKVSHALAEGLGVIACIGEKLDER
y AFTGENSVDQIKDVGAKWVILGHSERRSYFHEDDKFIADKTKFALGQGVGVILCIGETLEEK

h EAGITEKVVFEQTKVIADNVKDWSKVVLAYEPVWAIGTGKTATPQQAQEVHEKLRGWLKSNV
m EAGITEKVVFEQTKVIADNVKDWSKVVLAYEPVWAIGTGKTATPQQAQEVHEKLRGWLKSNV
y KAGKTLDVVERQLNAVLEEVKDWTNVVVAYEPVWAIGTGLAATPEDAQDIHASIRKFLASKL

h SDAVAQSTRIIYGGSVTGATCKELASQPDVDGFLVGGASLKPEFVDIINAKQ
m NDGVAQSTRIIYGGSVTGATCKELASQPDVDGFLVGGASLKPEFVDIINAKQ
y GDKAASELRILYGGSANGSNAVTFKDKADVDGFLVGGASLKPEFVDIINSRN
```

V-type proton ATPase subunit G (VMA10)

>sp|O75348|VATG1_HUMAN V-type proton ATPase subunit G 1 OS=Homo sapiens GN=ATP6V1G1
>sp|Q9CR51|VATG1_MOUSE V-type proton ATPase subunit G 1 OS=Mus musculus GN=Atp6v1g1
>sp|P48836|VATG_YEAST V-type proton ATPase subunit G OS=Saccharomyces cerevisiae (strain ATCC 204508/S288c) GN=VMA10

```
Human MASQSQGIQQLLQAEKRAAEKVSEARKRKNRRLKQAKEEAQAEIEQYRLQREKE
Mouse MASQSQGIQQLLQAEKRAAEKVSEARKRKNRRLKQAKEEAQAEIEQYRLQREKE
Yeast M-SQKNGIATLLQAEKEAHEIVSKARKYRQDKLKQAKTDAAKEIDSYKIQKDKE

Human FKAKEAAALGSRGSCSTEVEKETQEKMTILQTYFRQNRDEVLDNLLAFVCDIRP
Mouse FKAKEAAALGSHGSCSSEVEKETREKMTVLQNYFEQNRDEVLDNLLAFVCDIRP
Yeast --LKEFEQKNAGGVGELEKKAEAGVQGELAEIKKIAEKKKDDVVKILIETVIKP
```

```
Human EIHENYRING
Mouse EIHENYRING
Yeast SAEVHINAL
```
